# Systematic review and meta‐analysis of genomic alterations in acral melanoma

**DOI:** 10.1111/pcmr.13034

**Published:** 2022-03-07

**Authors:** Natasa Broit, Peter A. Johansson, Chloe B. Rodgers, Sebastian T. Walpole, Nicholas K. Hayward, Antonia L. Pritchard

**Affiliations:** ^1^ 56362 Oncogenomics Group QIMR Berghofer Medical Research Institute Brisbane Queensland Australia; ^2^ Faculty of Medicine University of Queensland Brisbane Queensland Australia; ^3^ Genetics and Immunology Group University of the Highlands and Islands Inverness UK

**Keywords:** acral melanoma, genomics, meta‐analysis, systematic review

## Abstract

Acral melanoma (AM) tumors arise on the palms, soles, fingers, toes, and nailbeds. A comprehensive systematic meta‐analysis of AM genomic aberrations has not been conducted to date. A literature review was carried out to identify studies sequencing AM. Whole‐genome/exome data from 181 samples were identified. Targeted panel sequencing data from MSK‐IMPACT were included as a validation cohort (*n *= 92), and studies using targeted hot spot sequencing were also collated for *BRAF* (*n *= 26 studies), *NRAS* (*n *= 21), and *KIT* (*n *= 32). Statistical analysis indicated *BRAF*, *NRAS*, *PTEN*, *TYRP1*, and *KIT* as significantly mutated genes. Frequent copy‐number aberrations were also found for important cancer genes, such as *CDKN2A*, *KIT*, *MDM2*, *CCND1*, *CDK4*, and *PAK1*, among others. Mapping genomic alterations within the context of the hallmarks of cancer identified four components frequently altered, including (i) sustained proliferative signaling and (ii) evading growth suppression, (iii) genome instability and mutation, and (iv) enabling replicative immortality. This analysis provides the largest analysis of genomic aberrations in AM in the literature to date and highlights pathways that may be therapeutically targetable.

**Significance**

This study is the first meta‐analysis and systematic review of acral melanoma genomics published to date. Our study aggregates genomic data from numerous studies, to present an in‐depth analysis of genomic aberrations and altered signaling pathways in acral melanoma. We contextualize these alterations within the context of the hallmarks of cancer and identify several components frequently altered, including those involved in proliferative signaling, genome instability, and replicative immortality.

## 1 INTRODUCTION

Acral melanoma (AM) occurs on glabrous skin, the non‐hair‐bearing skin of the volar surfaces of the extremities, including palms, soles, fingers, toes, and nailbeds (subungual). The genomic aberrations in AM differ from the other subtypes of cutaneous melanoma (CM; nodular, lentigo maligna, and superficial spreading), notably in number and signatures of mutations, and the frequency of chromosomal rearrangements, such as copy‐number alterations (CNAs) and structural variants (SV) (Hayward et al., 2017).

The etiology of AM is unclear, and mutational signatures associated with ultraviolet radiation (UVR) exposure are detected less frequently in AM than in CM (Hayward et al., 2017). AM with a UVR signature usually occurs in a subungual primary site (Newell et al., 2020). It is suspected that some AM may be induced by trauma, as they commonly arise in regions of physical stress (Costello et al., 2017; Elder et al., 2020; Jung et al., 2013).

Five‐year and ten‐year disease‐specific survival is significantly worse in stage‐matched AM *vs*. CM of the extremity (Bello et al., 2013; Bradford et al., 2009).

While studies identifying genomic changes in AM have provided insight into the alterations contributing to tumorigenesis, they have generally included a small number of samples, due to the rarity of AM. Now that several studies have been published it is timely to perform a meta‐analysis to identify the key alterations driving AM tumorigenesis (Berger et al., 2012; Cancer Genome Atlas, 2015; Furney et al., 2014; Hodis et al., 2012; Krauthammer et al., 2012; Liang et al., 2017; Lim et al., 2020; Newell et al., 2020; Snyder et al., 2014).

## 2 METHODS

The methods are briefly summarized here, and further details are provided in the Methods S1.

### 2.1 Data acquisition

#### 2.1.1 Main cohort studies

Whole‐genome and whole‐exome sequenced samples from fresh‐frozen (FF) tissue with matched normal DNA were included in the main cohort (Table 1). Ten studies were identified that matched these criteria (Berger et al., 2012; Cancer Genome Atlas, 2015; Furney et al., 2014; Hayward et al., 2017; Hodis et al., 2012; Krauthammer et al., 2012; Liang et al., 2017; Lim et al., 2020; Newell et al., 2020; Snyder et al., 2014). Data were accessed from cBioPortal (cbioportal.org/) for some studies (Berger et al., 2012; Cancer Genome Atlas, 2015; Hodis et al., 2012; Krauthammer et al., 2012; Liang et al., 2017; Snyder et al., 2014). Data from the remaining studies were downloaded from the supplementary tables of each respective manuscript (Furney et al., 2014; Hayward et al., 2017; Lim et al., 2020; Newell et al., 2020). Duplicate samples were identified and excluded (Methods S1).

**TABLE 1:** Number of samples included in each cohort, split by genomic aspect, detailing the study from which the data were derived

Aberration type	Number of samples	References
Main cohort (FF; WGS/WES)		
Single‐nucleotide variants and small insertions–deletions	181	(Berger et al., 2012; Cancer Genome Atlas, 2015; Furney et al., 2014; Hodis et al., 2012; Krauthammer et al., 2012; Liang et al., 2017; Lim et al., 2020; Newell et al., 2020; Snyder et al., 2014)
Copy‐number aberrations	125	(Liang et al., 2017; Newell et al., 2020; Snyder et al., 2014)
Structural variants	87	(Newell et al., 2020)
**Validation Cohort** (FFPE; Targeted Panel)		
Single‐nucleotide variants and small insertions–deletions	92	(Zehir et al., 2017) (Liang et al., 2017; Newell et al., 2020; Snyder et al., 2014)
Copy‐number aberrations		
Structural variants		
Targeted hot spot panel		
*BRAF*	Exon 11 (*n *= 944) Exon 15 (*n *= 1207)	(Abu‐Abed et al., 2012; Ashida et al., 2009; Borkowska et al., 2020; Carvajal et al., 2011; Cirenajwis et al., 2017; Colombino et al., 2013; Comodo‐Navarro et al., 2020; Curtin et al., 2006; Dai et al., 2013; Dika et al., 2020; Handolias et al., 2010; Hilke et al., 2020; Jin et al., 2013; Kang et al., 2016; Kong et al., 2011; Lin et al., 2013; Minor et al., 2012; Moon et al., 2018; Niu et al., 2013; Oyama et al., 2015; Puntervoll et al., 2014; Schoenewolf et al., 2012; Sheen et al., 2020; Shi et al., 2019; Shim et al., 2017; Terada, 2010; Torres‐Cabala et al., 2009; Yeh et al., 2019; Yun et al., 2011; Zaremba et al., 2019; Zebary et al., 2013; Zou et al., 2020) (Abu‐Abed et al., 2012; Akslen et al., 2008; Ashida et al., 2009; Borkowska et al., 2020; Carvajal et al., 2011; Choi et al., 2013; Cirenajwis et al., 2017; Colombino et al., 2013; Comodo‐Navarro et al., 2020; Curtin et al., 2006; Dai et al., 2013; Dika et al., 2020; Gao et al., 2018; Handolias et al., 2010; Hilke et al., 2020; Jin et al., 2013; Kang et al., 2016, 2018; Kong et al., 2011; Lang & MacKie, 2005; Lin et al., 2013; Minor et al., 2012; Moon et al., 2018; Niu et al., 2013; Oyama et al., 2015; Puig‐Butille et al., 2013; Puntervoll et al., 2014; Schoenewolf et al., 2012; Sheen et al., 2016, 2020; Shi et al., 2019; Shim et al., 2017; Si et al., 2012; Terada, 2010; Torres‐Cabala et al., 2009; Uhara et al., 2014; Yeh et al., 2019; Yun et al., 2011; Zaremba et al., 2019; Zebary et al., 2013; Zou et al., 2020)
*NRAS*	Exon 2 (*n *= 1065) Exon 3 (*n *= 1067)	
*KIT*	Exon 9 (*n *= 1059) Exon 11 (*n *= 1443) Exon 13 (*n *= 1443) Exon 17 (*n *= 1270) Exon 18 (*n *= 1035)	

#### 2.1.2 Validation cohort: targeted gene panel

Samples sequenced from formalin‐fixed paraffin‐embedded (FFPE) tissue with matched normal DNA from the AACR Project GENIE (2017) were included as a validation cohort (Table 1). The only study meeting these criteria was the Memorial Sloan Kettering‐Integrated Mutation Profiling of Actionable Cancer Targets (MSK‐IMPACT™; Zehir et al. (2017)). Version 8.0 was accessed via cBioPortal‐GENIE and downloaded via Synapse (synapse.org/genie). Validation cohort samples were sequenced on three generations of targeted gene panels (341 gene panel: *n *= 6; 410: *n *= 24; and 468: *n *= 62; Table 1, Table S1).

#### 2.1.3 Targeted hot spot sequencing

A PubMed search was conducted on March 30, 2020, to identify studies with a sequencing component targeting *BRAF*, *NRAS*, or *KIT*, as hot spot mutations in these genes are the most frequently studied in AM. These data were compiled to form the “targeted hot spot” cohort for *BRAF* (26 studies), *NRAS* (21 studies), and *KIT* (32 studies) (Table 1, Table S2).

### 2.2 Samples and anatomy

Primary sites reported in the main cohort (*n *= 181) were recoded into acral (*n *= 141) or subungual (*n *= 40). Further classification was made into upper (*n *= 24; hand/fingernails), lower (*n *= 143; foot/toenails), and not classified (*n *= 14) sites (Table S1). Site‐specific stratification on the validation cohort (*n *= 92) could not be performed as a specific primary site was not provided.

### 2.3 Bioinformatics

The data were analyzed as previously described (Broit et al., 2021) and summarized in the Methods S1. Briefly, the mutation calls from the multiple sources were merged and annotated using Ensembl Variant Effect Predictor (McLaren et al., 2016). For CNAs, only deletions (loss of heterozygosity (LoH, loss) and homozygous deletions (HD, deletion)) and high‐level copy gains (amplifications) were included in the reported frequency events (detailed in Table S1).

To perform the analyses and data visualization, a mutation annotation format (MAF) file was created using Funcotator (https://gatk.broadinstitute.orgl). MutSigCV version 1.3.5, accessed via the GenePattern Public Server (https://cloud.genepattern.org/), OncodriveFM (v1.0.3), and OncodriveCLUST (v1.0.0) (Gonzalez‐Perez et al., 2013), was used to identify significantly mutated genes (SMGs), and genes significant (*q* ≤ 0.1) with two or more tools are reported.

## 3 RESULTS

### 3.1 Genomics overview

**FIGURE 1:**
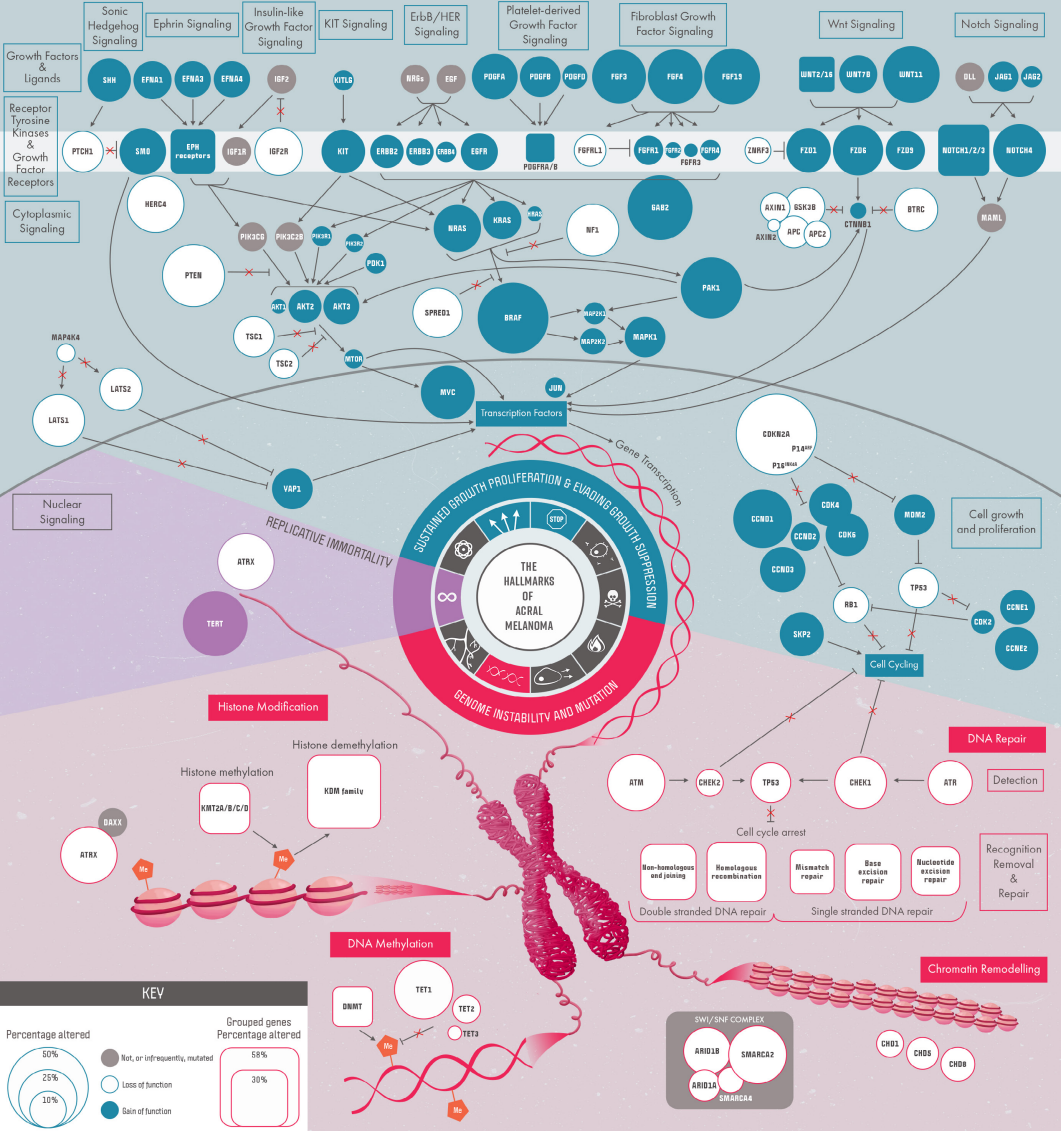
Overview of genomic alterations identified in acral melanoma. Shaded circles indicate alterations that are likely gain of function. Empty circles indicate alterations that likely lead to loss of function of the protein product. The prevalence of alterations is indicated by circle size, as detailed in the key.

An overview of the major functional genomic aberrations in the main cohort is provided in Figure 1. The frequency of genomic alterations found in the main cohort for mutations (*n *= 181), CNA (*n *= 125), and SV (*n *= 67) is reported in regular font. Aberrations present in the validation cohort (*n *= 92) are reported in italicized font (where absent, the gene was not present on the panel; Table 1). All genomic data and analysis are presented in Table S1.

### 3.2 Mutation signatures

**FIGURE 2:**
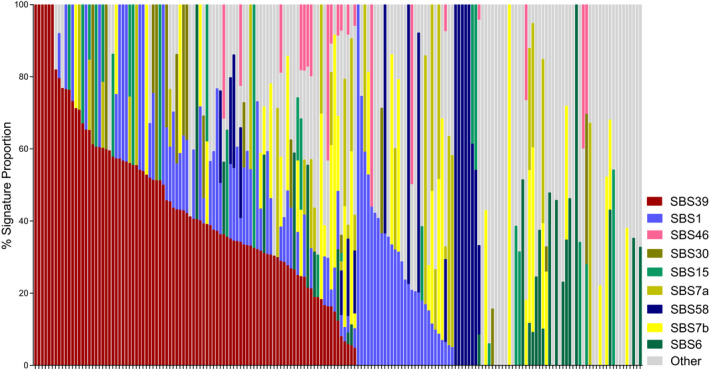
Mutation signatures. Signature contributions (%) detected in individual tumors. Signatures represented across the cohort commonly included SBS39 (unknown etiology), SBS1 (associated with deamination of 5‐methylcytosine to thymine), and SBS7a/b (associated with exposure to ultraviolet radiation)

The most recurrent mutation signature (Figure 2) detected was SBS39 (53% of samples), which is of unknown etiology. SBS1 was the next most common (46%) and is due to an endogenous mutational process initiated by spontaneous or enzymatic deamination of 5‐methylcytosine to thymine, generating G:T mismatches in double‐stranded DNA. SBS7, associated with UVR exposure, was detected in 34% of samples, with SBS7a and SBS7b being the most recurrent. SBS7 was the dominating signature (>50%) in 11% of samples. A larger fraction of the subungual samples (8/32 *vs*. 12/129 acral) were dominated by the SBS7 signature (Fisher's exact test: *p* = .0495).

Signatures SBS6 (13.8%) and SBS15 (13.8%), associated with defective DNA mismatch repair, were detected in a mutually exclusive pattern. SBS6 is associated with insertion–deletion mutations. Several tumors with SBS6 or SBS15 had an alteration in a mismatch repair gene (e.g., SBS6: *MSH5* p.R112Q, *MSH6* LoH; SBS15: *PMS1* p.W446X, *MSH5* p.A104T; and a sample with a HD in *MSH3*, *MSH4*, *MSH6*, and *PMS1*); however, not all samples had an identifiable aberration. Sixteen samples showed the SBS30 signature, which is associated with a deficiency in base excision repair ascribed to inactivating mutations in *NTHL*. One sample carrying this signature had HD of *NTHL1*.

### 3.3 Significantly mutated genes

**FIGURE 3:**
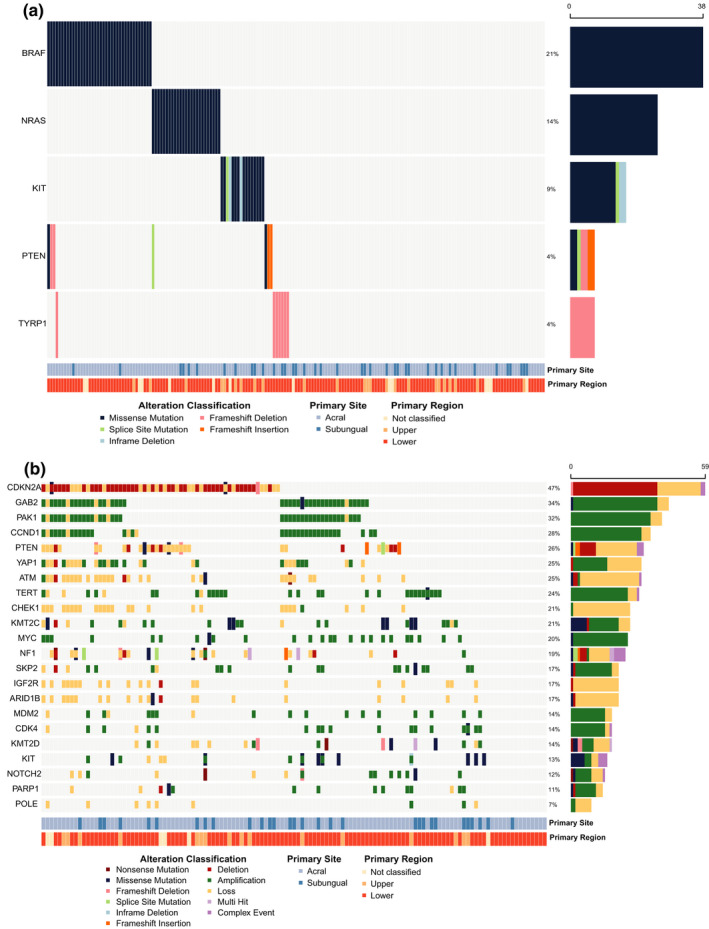
Significantly altered genes. (a) Significantly mutated genes were identified using MutSigCV, OncodriveCLUST, and OncodriveFML. Genes reported significant (*q* < 0.1) by two or more tools are displayed. (b) Genes with significant copy‐number aberrations reported in previous studies are summarized. CNAs are displayed as squares, and mutations and small indels, as higher rectangles. Recurrently amplified genes included *MDM2*, *CCND1*, *CDK4*, *SKP2*, *KIT*, *NOTCH2*, *GAB2*, *YAP1*, *MYC*, and *PAK1*. Regions of significant copy loss included *CDKN2A*, *NF1*, *PTEN*, *CBL*, and others involved in DNA repair and chromatin remodeling

*BRAF*, *NRAS*, *PTEN*, *TYRP1*, and *KIT* were SMGs, which were collectively altered in 88 of 181 tumors (Figure 3a). Details of the mutations present in these genes are provided in the following sections. Mutual exclusivity was observed between *BRAF*, *NRAS*, and *KIT*. Of particular note, *TYRP1* frameshift mutations, predominately p.N353Vfs*31 (6/7 tumors), were exclusively from one cohort (Newell et al., 2020); this variant (rs387906562) has been described as a pathogenic germline variant in oculocutaneous albinism type III (Chiang et al., 2009), but was only observed as a somatic mutation in these samples.

### 3.4 Notable copy‐number alterations

The genes significantly altered by CNA are briefly summarized here and in Figure 3b, with the specific frequencies noted within the following relevant sections.

Genes encoding proteins that function within the cell cycle pathway were notably affected by deletions (*CDKN2A*) and amplifications (*MDM2*, *CCND1*, *CDK4*, and *SKP2*). The genes encoding receptor tyrosine kinase (RTK) receptor *KIT* and growth factor receptor *NOTCH2* were amplified, while the genes encoding proteins that mitigate receptor signaling, CBL (a ligase), and IGF2R (decoy receptor for IGF2) were lost. Genes encoding components of cell signaling, including *GAB2*, *PAK1*, *YAP1*, and *MYC*, were significantly amplified. Loss of tumor suppressors *NF1* and *PTEN* were reported, and *TERT*, a gene encoding a telomere maintenance protein enabling replicative immortality, was amplified. Finally, several DNA repair genes were lost, including *POLE*, *PARP1*, *ATM*, and *CHEK1*, as were genes involved in chromatin remodeling, *ARID1B*, *KMT2D*, and *KMT2C*.

### 3.5 Key driver genes for other melanoma subtypes are altered in acral melanoma

Important driver genes in CM, uveal melanoma (UM), and mucosal melanoma (MM) were mutated in the AM main cohort (Table 2). CM is genomically classified into four subgroups based on the most prevalent and mutually exclusive mutated driver genes: *BRAF* with p.V600 (50%), *N*/*H*/*KRAS* with p.G12, p.G13, and p.Q61 (30%), *NF1* with loss‐of‐function (LoF; 10%) mutations, and a triple wild‐type group (10%) (Cancer Genome Atlas, 2015). These genes were also altered in AM, but at different frequencies (Table 2).

**TABLE 2:** Frequencies of gene alterations across melanoma subtypes

Gene	Acral melanoma (present study)		Cutaneous melanoma (Hayward et al., 2017)		Mucosal melanoma (Broit et al., 2021)		Uveal melanoma (Johansson et al., 2020)		Examples of other cancers with alterations^a^
	Mutation (*n *= 181)	CNA (*n *= 125)	Mutation (*n *= 140)	CNA (*n *= 140)	Mutation (*n *= 173)	CNA (*n *= 72)	Mutation (*n *= 103)	CNA (*n *= 103)	
*BRAF*	21%	Amp: 15.2%	55%	Amp: 26%	9.2%	Amp: 6.9%	–	–	Thyroid, colorectal adenocarcinoma
*NRAS*	13.8%	Amp: 2.4%	32.1%	Amp: 6.4%	8.6%	Amp: 2.7%	–	–	Cholangiocarcinoma, acute myeloid leukemia, thyroid carcinoma
*NF1* (LoF)	5.5%	HD: 3.2% LoH: 9.6%	12.1%	HD: 0.7% LoH: 15%	8.6%	HD: 1.4% LoH: 12.5%	–	LoH: 3.8%	Uterine corpus endometrial carcinoma, lung squamous cell carcinoma, ovarian serous cystadenocarcinoma
*CDKN2A*	1.6%	HD: 30.4% LoH: 16%	9.3%	HD: 22.1% LoH: 21.4%	–	HD: 25% LoH: 14.4%	–	HD: 1.0% LoH: 11.6%	Glioblastoma multiforme, head‐and‐neck squamous cell carcinoma, mesothelioma
*KIT*	8.7%	Amp: 5.6%	4.3%	Amp: 5.7%	19.1%	Amp: 15.3%	–	–	Testicular germ cell tumors, glioblastoma multiforme
*SPRED1* (LoF)	2.2%	HD: 3.2% LoH: 12.8%	2.1%	LoH: 13.6%	4%	HD: 4.2% LoH: 8.3%	–	LoH: 7.8%	Mesothelioma, uterine carcinosarcoma, lung adenocarcinoma
*PTEN* (LoF)	2.7%	HD: 5.6% LoH: 16.8%	6.4%	HD: 4.3% LoH: 27.8%	0.6%	HD: 5.5% LoH: 16.6%	–	LoH: 6.8%	Glioblastoma multiforme, prostate adenocarcinoma, lung squamous cell carcinoma
*ATRX* (Lo*F)*	1.6%	HD: 0.8% LoH: 20%	1.4%	LoH: 12.8%	3.5%	–	–	LoH: 11.7%	Brain lower grade glioma, sarcoma
*TERT (promoter mutations)*	9.2%	Amp: 20.8%	86%	Amp: 11.4%	9%	Amp: 16.6%	–	–	Lung squamous cell carcinoma, adrenocortical carcinoma, esophageal adenocarcinoma
*SF3B1*	0.5%	LoH: 3.2%	3.6%	LoH: 10.7%	8.1%	LoH: 2.7%	13.6%	LoH: 3.9%	Uterine corpus endometrial carcinoma, bladder urothelial carcinoma
*CTNNB1*	1.1%	Amp: 0.8%	6.4%	Amp: 6.4%	4.6%	Amp: 1.4%	–	–	Liver hepatocellular carcinoma, uterine corpus endometrial carcinoma
*BAP1*	0.5%	LoH: 8.8%	0.7%	LoH: 13.6%	1.7%	LoH: 4.2%	41.7%	LoH: 57.3%	Mesothelioma

Abbreviations: Amp, amplification; CNA, copy‐number aberration; HD, homozygous deletion; LoF, loss of function (truncating mutations).

^a^ Source: TCGA Pan‐Cancer Analysis (excluding all melanoma subtypes)—only selected cancers highlightedLoH: loss of heterozygosity.

**FIGURE 4:**
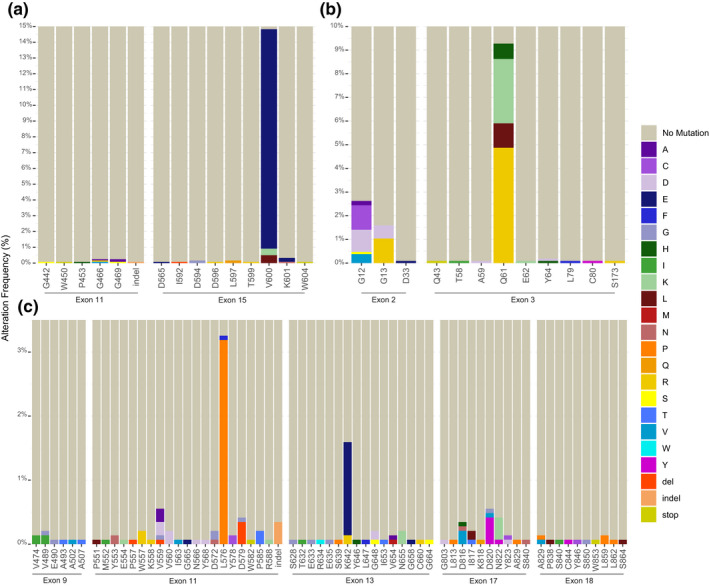
Targeted hot spot analysis. To identify recurrent mutations in well‐known melanoma genes *BRAF*, *NRAS*, and *KIT*, mutation reports from targeted sequencing studies were hand‐collated and the frequency of alterations was summarized by exon/codon. (a) Summary of mutations identified in *BRAF* exon 11 (*n *= 944) and exon 15 (*n *= 1207) mutations. (b) Summary of *NRAS* mutations in exon 2 (*n *= 1065) and 3 (*n *= 1067). (c). Summary of *KIT* mutations in exons 9 (*n *= 1059), 11 (*n *= 1443), 13 (*n *= 1443), 17 (*n *= 1270), and 18 (*n *= 1035). *KIT* single codon deletions indicated as “del.” Larger insertions–deletions are indicated as “indel.” Codon position is indicated along the x‐axis of each panel. Percentages expressed represent the frequency of alterations by exon. Colors are indicative of amino acid changes identified

*BRAF* was an SMG and the most recurrently mutated gene in AM (21.0%, *11.9*%), occurring at a lower frequency than in CM (55%) (Hayward et al., 2017), but higher than in MM (9.2%) (Broit et al., 2021; Table 2; Figure 3a; Figure S1a). The p.V600E mutation was the most recurrent *BRAF* mutation (80%; *91.0*%), which was confirmed in the targeted gene panel data (*n *= 168 of 192 mutations of 1207 samples assessed; Table S2a). Codon 600 was also altered by amino acid changes p.V600 M/K/L (Table S2a; Figure 4a). Collectively, the p.V600 variants are known as type I BRAF mutations, which comprised 82.5% of observed changes. Other codons were recurrently mutated, including p.D594, p.K601, p.G466, and p.G469, though these were infrequent (Tables S1 and S2a); of these, 15% were type II mutations. A single sample contained a type III BRAF mutation, which co‐occurred with a type III NRAS mutation. Amplifications of *BRAF* occurred more frequently in AM (15.2%) than in MM (6.9%), but less than in CM (26%), and they co‐occurred with p.V600E (5/7 co‐occurrences), p.G469E (1/7), and p.G464V (1/7) (Figure S1b). *BRAF* variants were rare in subungual tumors (2/40; p.D594G and p.V600E).

As in CM and MM, *NRAS* was the most frequently altered RAS gene (13.8%, *20.6*%), and mutations most often affected codon 61 and less frequently codons 12 and 13 (Figure 3a; Table S1). The targeted hot spot data identified several further rare *NRAS* mutations that were not present in the main/validation cohorts (Figure 4b; Table S2b). Mutations in *KRAS* (2.8%, *5.4*%) and *HRAS* (1.7%, *2.8*%) were less common and largely occurred in codons 12, 13, and 61 (Figure S1a; Table S1). Together, most *H*/*K*/*NRAS* variants occurred at these hot spots (94%). Amplifications of *NRAS* (2.4%) occurred at a similar frequency as in MM (2.7%); *KRAS* amplifications were also recurrent (8%, *9.7*%), but *HRAS* was not subject to CNA (Figure S1b).

As observed in CM and MM, *NF1* mutations (7.2%, *6.5*%) were mainly non‐clustering LoF mutations (Figure S1a) and loss also occurred by CNA (HD: 3.2%, *0*%; LoH: 9.6%, *2.4*%) and SV (2.3%) (Table 2; Figure S2a).

*KIT* was an SMG in AM, with mutations clustered within the juxtamembrane domain (exon 11) and the kinase domains (exons 13, 17). The most common COSMIC hot spot in *KIT* is p.D816, but these mutations were rare in AM (0.5%, *1.6*%). Rare activating mutations with supportive functional evidence (Ashida et al., 2009; Conca et al., 2009; Duensing et al., 2004; Ma et al., 1999) were p.L576P (1.6%, *0*%) and p.K642E (1.6%, *1.6*%), which are recurrent in MM but rare in CM (Figure S2a). Targeted hot spot sequencing confirmed p.L576P (*n *= 46/1443) and p.K642E (*n *= 21/1443) were recurrent in AM (Figure 4c, Table S2c). Other activating mutations, including p.D816V (Bodemer et al., 2010), p.D820Y (Ashida et al., 2009), and p.N822K (Duensing et al., 2004), were identified. In total, the frequency of hot spot mutations in AM (8.7%) is comparable to CM (~5%) but notably different to MM (19.1%; Table 2); subungual (10%) samples were comparable to acral (6.1%) samples. Amplifications of *KIT* were also less common in AM (5.6%, *3.3*%) or CM (4.3%) than in MM (15.3%; Table 2; Figure S2b). Co‐occurrence of mutation and amplification in AM was identified in four samples, two of which involved mutation p.K642E, which has been reported to co‐occur with amplification events (Curtin et al., 2006).

SPRED1 is a negative regulator of the mitogen‐activated protein kinase (MAPK) pathway (Wakioka et al., 2001). Function of SPRED1 was lost by various mechanisms, including mutation (LoF: 2.2%, *1.1*%), deletion (HD: 3.2%, *6.5*%; LoH: 12.8%, *1.1*%), and SV (1.5%) (Figure 1). Loss of *SPRED1* occurred at comparable rates in MM and CM (Table 2, Figures S1a, b).

PTEN acts as a critical negative regulator of PI3K signaling by removing the D3 phosphate from PIP3 to produce phosphatidylinositol 4,5 biphosphate (PIP2). *PTEN* was an SMG in AM, where it was frequently lost (LoF: 2.7%, *3.3*%; HD: 5.6%, *1.1*%; and LoH: 16.8%, *4.4*%). While the LoF mutation rates varied (6.4% and 0.6%), the frequency of CNAs was comparable to that in CM and MM (Table 2, Figures S1a, b).

ATRX acts with the histone chaperone DAXX to insert histone variant H3.3 (Nandakumar et al., 2017; Pritchard, 2019), and loss of ATRX leads to increased homologous recombination facilitating telomere destabilization and alternative lengthening of telomeres (ALT; Abedalthagafi et al., 2013; Dyer et al., 2017; Marinoni et al., 2014; Yuan et al., 2020). LoF mutations (1.7%) and copy loss (HD: 0.8%, *0*%; LoH: 20%, *0*%) were observed in AM at a similar frequency to CM, but are slightly more common in MM (3.7%), where they are associated with tumors of the lower anatomy (Broit et al., 2021) (Table 2).

Mutations in *CTNNB1* were recently reported as an SMG in MM (4.6%) (Broit et al., 2021) and are common in CM (6.4%) (Table 2). In AM, alterations to *CTNNB1* were less frequent (gain of function (GoF): 1.1%, *2.2*%; amplification: 0.8%; *0*%), with hot spot mutations (p.S33F, p.S37C, p. G34R), occurring in subungual tumors (Table 2).

*SF3B1* encodes a component of the spliceosome involved in pre‐mRNA splicing (Kesarwani et al., 2017) and has previously been identified as an SMG in UM (Furney et al., 2013; Harbour et al., 2013) and MM (Broit et al., 2021). In AM, mutations were infrequent (0.5%, *1.1*%) involving variants of unknown significance (VUS), not previously described in TCGA (Table 2).

LoF variants or HD in *BAP1* are commonly described in UM (Harbour et al., 2010) and infrequently in CM (Hayward et al., 2017); in AM, a LoF mutation (p.N308Qfs*90) was identified and LoH was infrequent (8.8%), occurring only in the main cohort (Table 2).

### 3.6 Altered cellular pathways in AM

The following sections detail genomic alterations observed in AM within (a) the hallmarks of cancer (Hanahan & Weinberg, 2011) as a contextual framework and (b) the cellular pathways these changes affect. Figure 1 summarizes these data, providing an overview of the impact of these changes. Four major hallmark components were notably altered in AM, as described in the following sections.

#### 3.6.1 Hallmarks: (1) sustained proliferative signaling and (2) evading growth suppression—ligands and receptors

Growth factors signal through receptors to activate a variety of cell proliferation pathways (Figure 1). Genes encoding RTKs generally had low‐frequency mutations, but were regularly altered by copy‐number changes, as further detailed below (see Figure S2a,b). The most notably affected RTK was KIT, as previously described; the gene encoding the ligand of KIT, KITLG, was occasionally amplified (2.4%). Two genes were significantly specifically affected by CNA in the RTK pathway. *IGF2R*, encoding a receptor that attenuates the signal from the growth factor IGF2, was significantly lost in AM (HD: 0.8%; LoH: 16%), and this may result in increased IGF2 signaling (Brown et al., 2009). *CBL*, encoding an E3 ubiquitin ligase targeting degradation of RTK (Brand et al., 2014; Joazeiro et al., 1999), was significantly lost (LoH: 18.4%, *0* *%*; HD: 0%, *1.1* *%*).

##### Growth factor signaling receptors

There are several members of growth factor receptor families that are altered in AM, mainly through CNA (Figure 1; Figure S2a,b), which have shown functional relevance in other cancers (e.g., Chen et al., 2016; Donnem et al., 2008; Slamon et al., 1987; Wei et al., 2021). These include the receptors *PDGFRA*/*B* (7.2%, *2.2*%), *EGFR* (8.8%, 0%), *ERBB2* (HER2; 6.4%, *3.3*%), *ERBB3* (HER3; 4.8%, *3.3*%), *FGFR1* (5.6%, *1.1*%), and *FGFR4* (2.4%, *1.1*%). FGFRL1 can function as a decoy receptor binding FGF ligands to sequester away from FGFRs (Trueb, 2011); copy loss of the *FGFRL1* gene was found (7.2%). VUS in growth factor signaling receptors was uncommon; however, two mutations (p.G316E in *FGFR2* and p.V642A in *FGFR3*) reside adjacent to previously described recurrent mutations (Chesi et al., 2001; Greulich & Pollock, 2011; Webster et al., 1996), which would be interesting to assess for functional impact. Ligands were uncommonly affected, aside from amplification of the FGF gene cluster on 11q13: *FGF19* (22.4%), *FGF4* (21.6%), and *FGF3* (20.0%).

##### Eph receptor family

Erythropoietin‐producing hepatoma (EPH) receptor subfamily is the largest among the RTKs. The ephrin receptors EPHA and EPHB differ by the way the two subgroups are tethered to the plasma membrane (Liang et al., 2019). Genes of the *EPHA* receptor class were amplified in 21.6% of tumors, while those of the *EPHB* class were classified in 12%; genes encoding several ephrin ligands, including *EFNA1*‐*5* and *EFNB2*‐*3*, were also amplified (total: 17.6%) (Figure 1).

#### 3.6.2 Hallmarks: (1) sustained proliferative signaling and (2) evading growth suppression—signaling pathways

##### MAPK Pathway

Upon RTK activation, GAB2 interacts with receptors serving as a gateway for activation of the downstream signaling cascade, including RAS‐RAF‐MEK‐ERK, and RAC/JNK, STATs and AKT; amplification has been shown to directly influence proliferation, and cell cycle progression in breast (Bocanegra et al., 2010) and ovarian (Dunn et al., 2014) cancer. *GAB2* was one of the most frequently amplified genes (29.6%) in AM (Figure S1b). Upon signal, RAS proteins can be activated via displacement of GDP and binding of GTP, initiating signaling through a protein cascade to stimulate proliferation. As previously described, aberrations in the RAS proteins are important driver events in AM and downstream of RAS, the RAF serine threonine kinases signaling though the MAPK pathway, including the proteins encoded by the SMGs *BRAF*, *SPRED1* and *NF1* (Figure 1; Table 2).

Downstream of BRAF, MEK1/2 (*MAP2K1*/*MAP2K2*), and ERK1/2 (*MAPK3*/*MAPK1*) activate multiple cytoplasmic substrates and transcription factors (Figure 1). Mutations in MEK1/2 were infrequent (*MAP2K1*: 1.1%, 0%; *MAP2K2*: 1.6%, *1.1*%); however, the observed *MAP2K1* mutation p.C125S has been described as homologous to p.C121S in CM, to confer resistance to MEK inhibition (Van Allen et al., 2014). Amplifications occurred in *MAP2K1* (2.4%, *2.2*%), *MAP2K2* (3.2%, *0*%), and *MAPK1* (12%, *3.3*%), but no aberrations were identified in *MAPK3* (Figures S1a,b).

##### PTEN‐PI3K‐AKT signaling pathway

RTKs and RAS proteins can activate the PI3K pathway, leading to an increase in the cellular levels of phosphatidylinositol 3,4,5 triphosphate (PIP3) (Figure 1). Tumor suppressor gene *PIK3R1* (p85‐α) was lost (HD: 0.8%; LoH: 6.4%), whereas oncogene *PIK3R2* (p85‐β) was amplified (2.4%) (Vallejo‐Diaz et al., 2019). PIP3 binds to and activates protein kinases that were amplified in AM: *PDK1* (3.2%) and AKT, which has three isoforms encoded by: *AKT1* (1.6%, *0*%), *AKT2* (8.0%, *3.3*%), and *AKT3* (8.8%, *1.1*%). An activating mutation in *AKT3* (p.E17K) previously described in breast, ovarian, and colorectal cancer (Davies et al., 2008) was present in one sample. PTEN is a critical negative regulator of PI3K signaling and, as described previously, is an SMG in AM. Downstream of AKT, mTOR (amplification: 1.6%, *1.1*%) is activated to initiate cellular proliferation, which can be inhibited by TSC1 (LoH: 12.0%, *0*%, HD: 0%, *1.1*%) and TSC2 (LoH: 4.0%, *0*%, HD: 1.6%, *0*%); these alterations likely promote signaling through this pathway (Figures S1a,b).

##### Wnt signaling

The Wnt signaling pathway is activated when a member of the WNT family of ligands binds to the extracellular domain of the Frizzled (FZD) receptor family (Figure 1). While mutations were rare in genes encoding Wnt ligands (total: 2.2%), amplifications were more frequent, including *WNT2* (13.6%), *WNT7B* (10.4%), *WNT11* (23.2%), and *WNT16* (14.4%), with *WNT16* and *WNT2* amplifications occurring together in 17 of 18 samples (both are on 7q31). Mutations were rare in the FZD receptor genes, but amplifications were frequent, including *FZD1* (13.6%), *FZD6* (17.6%), and *FZD9* (11.2%). *ZNRF3* (LoH: 4.8%) is involved in negative regulation of Wnt signaling by regulating the membrane levels of FZD and LRP (Hao et al., 2012) (Figures S1a,b).

Activation of this signaling pathway can induce several intracellular signaling transduction cascades, including the canonical (Wnt/β‐catenin‐dependent) or the non‐canonical (β‐catenin‐independent) pathway. The β‐catenin destruction complex is made up of a large multiprotein assembly with the core complex including proteins encoded by *APC* (LoF: 2.6%, *1.1*%; LoH: 5.6%, *0*%), *APC2* (HD: 0.8%; LoH: 4.0%), *AXIN1* (LoH: 6.4%), and *AXIN2* (LoF: 0.5%, *0*%). As previously described, infrequently in AM, β‐catenin (*CTNNB1*) is affected by hot spot variants, resulting in protein activation (Figures S1a,b).

##### Notch Signaling

Notch signaling is involved in developmental and post‐developmental contexts, such as tissue homeostasis and maintenance of stem cells in adults (reviewed in Yamamoto, 2020). In the Notch family of receptors, amplifications in genes *NOTCH1–3* were infrequent individually (9.6% total), but *NOTCH4* amplifications were more common (19.2%) (Figure 1). Mutations in receptors were also uncommon (total: 3.8%, *6.6*%); however, truncating mutations were observed in *NOTCH2*, of which two were early‐truncating (p.P6Rfs*27) (Yamamoto, 2020) and two were late‐truncating mutations (p.I2304Lfs*2; p.W2436*). Similar late‐truncating mutations have been described in hematological malignancies (Kiel et al., 2012; Weng et al., 2004), and as they result in the removal of degradation motifs that regulate protein stability, are proposed to be GoF (Aster et al., 2011; Chiang et al., 2006). Two fusion events in Notch receptors were reported in MSK‐IMPACT data: NOTCH2‐PRMT6 and NOTCH3‐ILVBL. Amplification of genes encoding mammalian Notch receptor ligands *JAG1* (5.6%) and *JAG2* (1.6%) was also present (Figures S1a,b).

##### Sonic Hedgehog signaling

Activation of the canonical sonic hedgehog (SHH) pathway can occur via SMO, a GPCR‐like (g‐protein coupled receptor) transmembrane protein. Upon binding of its ligand, SHH (amplified: 13.6%) and SMO (amplified: 14.4%; *1.1*%) activation can lead to downstream signaling, regulated by Patched (PTCH1; LoH: 12.0%, *0*%), which is inhibitory (pathway reviewed in Carballo et al., 2018; Figure 1). HERC4, an E3 ligase shown to negatively regulate SMO in a drosophila model (Sun et al., 2019), was lost in a number of samples (HD: 2.4%; LoH: 20.8%; Figures S1a,b).

##### Other notable genes involved in signaling

PAK1 (amplification: 28%, *21.7*%) serves a multitude of roles and functions as a central node across several signaling pathways, including the PI3K/AKT (Higuchi et al., 2008; Mao et al., 2008) and MAPK (Shrestha et al., 2012) pathways (Figure 1). YAP1 is a transcriptional coactivator (Stein et al., 2015) found to be significantly amplified in both cohorts (12%, *13*%; Figure S1b).

#### 3.6.3 Hallmarks: (1) sustained proliferative signaling and (2) evading growth suppression—cell cycle

Following the cell signaling cascades initiated by the ligand/receptors, activity within the nucleus completes the proliferation response (Figure 1). Several genes involved in the cell cycle pathway were altered in AM, usually by CNA (Figure S3a,b).

The loss of *CDKN2A* (HD: 30.4%, *21.7*%; LoH: 16%, *0*%; LoF: 0.5%, *1.8*%) removes regulatory mechanisms of cell cycle progression via two critical encoded protein products p14^ARF^ and p16^INK4A^. Mutations were uncommon (1.1%; *1.1*%) (Figure S3a), but each disrupts protein function (Ruas et al., 1999; Yarbrough et al., 1999). Isoform p14^ARF^ acts as an inhibitor of MDM2, an E3 ubiquitin ligase targeting p53 for ubiquitination and degradation. *MDM2* was frequently amplified (12%, *12.9*%), which could result in reduced levels of p53 expression (Oliner et al., 2016) (Figure S3a). Some amplification events in *MDM2* (6/15) co‐occurred with loss of *CDKN2A*. Isoform p16^INK4A^ interacts with the RB1 pathway, by binding to CDK4 (amplified: 12.8%, *17.4*%) and CDK6 (amplified: 12.8%, *1.1*%), to prevent RB1 phosphorylation and activation of target genes (Figure S3b). Upon mitogenic stimulation, D‐type cyclins *CCND1* (amplification: 24.8%, *20.6*%), *CCND2* (amplification: 5.6%, *5.5*%), and *CCND3* (amplification: 14.4%, *2.2*%) can bind to CDK4 and CDK6 to form complexes that phosphorylate Rb (Figure S3b). RB1 phosphorylation partially depresses activity of E2F family of transcription factors, facilitating expression of E2F target genes, including those encoding E‐type cyclins, which were both recurrently amplified (*CCNE1*: 7.2%, *2.2*%; *CCNE2*: 13.6%; Figure S3b). Cyclin E then binds to and activates CDK2 (amplification: 4%), which hyper‐phosphorylates RB1, further increasing the expression of E2F target genes. *RB1* was lost in several samples (LoH: 8.8%, *0*%; HD: 0.8%, *1.1*% Figure S3b).

Aberrations disrupting the function of p53 were present in AM (LoF: 3.3%, *1.1*%; LoH: 9.6%, *0*%; HD: 0%, *1.1*%; Figure S3a). The missense variants clustered in the hot spot central DNA binding domain, which are frequently mutated in cancer (p.R248W: *n *= 739; p.R273C: *n *= 707 in somatic IARC TP53 database) and are classified as “contact” mutations, for which the overall architecture of the DNA binding domain is retained, but there is loss of critical DNA contact (Cho et al., 1994; Olivier et al., 2010).

*SKP2* (amplified: 12.8%; Figure S3b) promotes S phase of the cell cycle, via degradation of target proteins that halt progression, including the CDK inhibitor p27, and increased expression drives proliferation (Gstaiger et al., 2001). MYC (amplified: 19.2%, *5.4*%; Figure S3b) acts as a stimulatory molecule for entering the cell cycle after signaling from MAPK, P13/AKT, and WNT pathways (Gabay et al., 2014). *JUN* (amplified: 2.4%, *2.2*%) encodes c‐Jun, which, in combination with c‐Fos, forms the AP‐1 early‐response transcription factor, required for cell cycle progression. Overexpression of c‐Jun has been shown to lead to an aggressive tumor phenotype in liposarcomas (Mariani et al., 2007).

#### 3.6.4 Hallmark: (3) genome instability and mutation

##### DNA repair pathways

The cell cycle can also be halted via the DNA damage repair pathway, and loss of function of these processes can remove these brakes. The proteins encoded by *ATR* (HD: 0.8%, *1.1*%; LoH: 12.8%, *0*%) and *ATM* (HD: 1.6%, *1.1*%; LoH: 21.6%) are activated in response to single‐ or double‐stranded DNA breakages, respectively. ATR and ATM activate CHEK1 (LoH: 20%, *0*%; HD: 0%, *1.1*%) and CHEK2 (LoH: 4%; Figure S3b), respectively, to halt the cell cycle via activation of p53, which inhibits cell cycle progression at the G1/S regulation point to allow for DNA damage repair to occur. Tumors with alterations in *ATM* or *ATR* (28.9%) displayed co‐occurring copy loss of both genes, with one tumor carrying HD of both genes. *CHEK1* was frequently lost with *ATM* (*n *= 23/25; Fisher's exact test: *p* < 0.0001) and *ATR* (*n *= 10/25; Fisher's exact test: *p* = 0.0001; Figure S3b).

##### Single‐stranded DNA repair

There are three main pathways of single‐stranded DNA repair (Figure 1), which comprises many components (reviewed by Altieri et al., 2008; Chatterjee & Walker, 2017), some of which are likely functionally aberrant in AM. Mutations were uncommon, but frequent CNAs were present (Figure S4a,b).

The nucleotide excision repair pathway removes bulky lesions caused by UVR or damage from chemotherapeutic agents. The UV‐DDB complex, including *DDB1* (LoH: 10.4%) and *DDB2* (LoH: 8.0%; Figure S4b), facilitates binding of XPC to UVR‐induced lesions, and experimental evidence suggests its knockdown increases tumorigenic potential (Roy et al., 2013) and reduces overall survival (Bommi et al., 2018) in various cancers.

In the base excision repair (BER) pathway, damaged bases are removed by a class of enzymes called DNA *N*‐glycosylases, of which several were lost, including *NEIL1*‐*3* (HD: 1.6%; LoH: 14.4%), *OGG1* (LoH: 6.4%), and *NTHL1* (HD: 1.6%; LoH: 4%; Figure S4b). The major polymerase responsible for inserting the correct nucleotide in BER is POLβ (LoH: 5.6%)(Carter & Parsons, 2016). Several proteins involved in long‐patch BER (Altieri et al., 2008; Carter & Parsons, 2016) were also altered, including POLD and POLE (combined LoH: in 20% samples) and FEN‐1 (LoH: 10.4%; Figure S4).

The mismatch repair pathway corrects errors introduced during DNA replication, where the MutSα heterodimer (MSH2‐MSH6) recognizes small base mismatches, and the MutSβ heterodimer (MSH2‐MSH3) recognizes larger errors. There was loss of the genes encoding these components in several tumors (LoF: 1.1%, *1.1*%; HD: 1.6%, *0*%; LoH: 8.8%, *0*%; Figure S4a,b). Following mismatch recognition, MutL complexes are recruited to the repair site, including the heterodimer of MLH1 (HD: 0.8%, *0*%; LoH: 5.6%, *0*%) and PMS1 (LoF: 0.5%, *0%*; HD: 0.8%, *0*%, LoH: 1.6%, *0*%; Figure S4a,b).

##### Double‐stranded DNA break repair

Double‐stranded breaks can lead to chromosomal aberrations and play an important role in tumorigenesis. Two major repair pathways resolve breaks: homologous recombination (HR) and non‐homologous end joining (NHEJ) (comprehensively reviewed by Chatterjee & Walker, 2017), some components of which are likely functionally altered in AM (Figure 1).

In NHEJ, 53BP1 (TP53BP1; HD: 0.8%, *6.4*%; LoH: 13.6%, *0*%) plays an important regulatory role by recruiting components of the pathway to the break site and polymerases POLμ (*POLM*; LoH: 2.4%) or POLλ (*POLL*; HD: 0.8%, LoH: 13.6%) fill the gaps (Figure S4).

In HR, the MRE11 (HD: 0%, *2.2*%; LoH: 14.4%, *0*%)–RAD50 (LoF: 0%, *1.1*%; HD: 0%, *1.1*%; LoH: 4.8%, *0*%)–NBS1 (*NBN*; LoH: 0.8%, *0*%) complex initiates this pathway at the double‐stranded break (Figure S4a,b). The E3 ligases ubiquitinate H2AX, which serves as a docking site for 53BP1 and BRCA1 (LoH: 7.2%). RPA (LoH: 8.8%) and RAD51 (HD: 0.8%, *5.4*%; LoH: 12.8%, *0%*) are recruited to prime the DNA, which is aided by BRCA2 (HD: 0%, *1.1*%; LoH: 7.2%, *0*%) and PALB2 (LoH: 4.8%, *0*%) (Figure S4b).

### 3.7 Chromatin modifications

Modifications to chromatin and DNA structure in tumors lead to altered epigenetic states and changes to chromatin access by regulatory proteins, contributing to tumorigenesis. Chromatin regulatory factors (CRFs) control chromatin structure and DNA modifications; there are three main classes of CRF that we discuss in the context of AM below (Figure 1).

#### 3.7.1 ATP‐dependent chromatin remodeling complexes

There are four classes of ATP‐dependent chromatin remodeling complexes, but the majority of components were only rarely altered in AM. The loss of the switch/sucrose non‐fermentable (SWI/SNF) complex leads to increased H3K27 methylation and tumor cell cycle progression (Wilson et al., 2010). Components of this complex were lost in AM, including *ARID1A* (LoH: 6.4%, *0*%), *ARID1B* (HD: 0.8%, *2.2*%; LoH: 15.2%, *0*%), *SMARCA2* (HD: 0.*8*%, LoH: 22.4%) and *SMARCA4* (LoH: 4%, *0*%) (Figure S5b). Loss of *ATRX* in AM is described above (Table 2; Figure S5a).

#### 3.7.2 Histone tail modifiers

Lysine methyltransferases (KMT) leave methylation marks on histone tails. Loss of *KMT2A*/*2B*/*2C*/*2D* (LoF: 3.3%, *3.2%*; HD: 0.8%, *1.6*%; LoH: 23.2%, *0*%; Figure S5a,b ) results in aberrant methylation control leading to overexpression of a wide variety of target genes (Rao & Dou, 2015). EZH2 is an epigenetic modifier that suppresses gene expression via histone methylation. Overexpression of EZH2 is found in different types of cancer, and recurrently, amplification was present in AM (14.4%, *0.8*%). The single VUS in *EZH2* (p.F642L) was adjacent to the melanoma hot spot (p.Y646F) (Souroullas et al., 2016). Members of the lysine demethylase (KDM) family were often mutated (LoF: 1.1%; VUS: 8.3%; *2.2*%; *note*: *Not all members were included in MSK*‐*IMPACT*), and one or more members were lost in 50% of tumors (*note*: *KDM5C and KDM5D were excluded*, *as they reside on sex chromosomes*), which can result in the loss of control of gene silencing by methylation (D'Oto et al., 2016; Figure S5a).

#### 3.7.3 DNA methyltransferase and demethylases

Gene promoter methylation is modified by members of the DNMT and TET family of proteins. Within the TET family, *TET1* (HD: 1.6%, *0*%; LoH: 22.4%, *0*%), *TET2* (HD: 0.8%, *0*%; LoH: 4.8%, *0*%), and *TET3* (LoF: 0.5%; LoH: 0.8%) were lost. Mutations in members of the DNMT family were uncommon, but loss via CNA was present in 15% of tumors (Figure 1, Figure S5a,b).

### 3.8 Hallmark: (4) enabling replicative immortality

*TERT* is a component of the telomerase complex that lengthens the telomeres of DNA strands to allow a cell to exceed the Hayflick limit and become potentially immortal. *TERT* is often a target of genomic change to stabilize telomeres through the many cycles of replication that cancer cells undergo. The *TERT* promoter is frequently mutated in CM (Table 2), but less frequently (9.2%) in AM (Newell et al., 2020). However, *TERT* was frequently amplified in AM (20.8%; *15.2*%; Figure 3b). As previously described, ATRX loss occurs in AM and can lead to the ALT, which also stabilizes telomeres.

#### 3.8.1 Clinically actionable biomarkers

Cancer genome interpreter (CGI) was used to identify biomarkers of drug response. Briefly, a select few examples are listed here. For full analysis and specific genes, see Table S1.

##### Receptor tyrosine kinases

As detailed (Figure 1, and above), a number of RTK classes were altered in AM.

The high frequency of *KIT* mutations makes it an attractive drug target, and there are a broad range of inhibitors targeting the receptor; despite this, currently no KIT inhibitor is clinically approved for use in AM.

As genomic amplification of *EGFR* occurred in AM (8.8%), it may be a good therapeutic candidate. Cetuximab (EGFR monoclonal antibody) is FDA‐approved for EGFR‐expressing tumors in head‐and‐neck cancer and colorectal cancer. Cetuximab is not indicated in EGFR expression colorectal cancer with a Ras‐mutant due to treatment‐related toxicity; *EGFR* amplification co‐occurred with an *NRAS* (p.G12S) mutation in a single tumor.

Amplifications were present in ERBB2 (6.4%), which can be targeted with inhibitors lapatinib and pertuzumab. A single tumor (without amplification) carried p.L755S, a known lapatinib resistance mutation due to conformational change to the protein (Bose et al., 2013).

*FGFR1*/*2 and FGF3*/*4*/*19* amplifications were common in AM. The ligands–receptors can be targeted with modest efficacy with lucitanib, a non‐specific FGFR1/2 inhibitor (Soria et al., 2014).

##### Cell signaling

AKT (collectively amplified in 16%) is an attractive drug target in AM. The *AKT1*‐activating mutation p.E17K responds to allosteric AKT inhibitor AZD5363 (Hyman et al., 2017); a p.E17K mutation was present in *AKT3*; however, the effect of AZD5363 in this context is unknown. An ATP‐competitive AKT kinase small‐molecule inhibitor GSK‐690693 inhibits phosphorylation in an *in vitro* overexpression model (Banerji et al., 2012), which should be tested in AM with AKT3 amplification.

Everolimus (mTOR inhibitor) was highlighted for several alterations, including *PTEN*, *CTNNB1*, and *TSC1*/*2*. Everolimus was associated with a longer progression‐free survival in prostate tumors with deregulated PTEN (NCT00976755; Templeton et al., 2013). Patients with endometrial cancer harboring *CTNNB1* mutations responded well to everolimus (NCT01068249; Slomovitz et al., 2015), and tumors with TSC1/2 loss could also be targeted by mTOR inhibition (Bissler et al., 2013; Wagner et al., 2010).

##### Cell cycle pathway

Given the high proportion of AMs with alterations in cell cycle pathway machinery, this is an attractive target; however, to date few compounds have progressed to clinical trial. A prospective phase II clinical trial to determine the efficacy of palbociclib in AM tumors that have *CDK4* and/or *CCND1* amplification and/or *CDKN2A* loss is currently underway (Clinical Trials ID: NCT03454919). Ribociclib (LEE011) was tested in phase II trials in patients with solid tumors and/or hematologic malignances with CDK4/6 pathway‐activated tumors (NCT02187783), but most patients succumbed to progressive disease.

## 4 DISCUSSION

While the gold‐standard technical approach for comprehensive tumor sequencing is WGS, from FF tumors with matched germline, the associated costs and difficulty in gaining access to clinical tissue specimens for research purposes can be prohibitive. Techniques such as WES, targeted panel, and Sanger sequencing may also be applied on both FF and FFPE tissues, and these give interesting insight into genomic alterations of tumors. In this study, we included WGS/WES in the main cohort, while targeted panel and focused Sanger sequencing from FFPE tissue were included as separate validation cohorts.

Studies using WGS and WES often presented smaller cohort sizes due to the rare nature of the cancer (Berger et al., 2012; Cancer Genome Atlas, 2015; Hayward et al., 2017; Hodis et al., 2012; Krauthammer et al., 2012; Liang et al., 2017; Lim et al., 2020; Newell et al., 2020). It is therefore timely to aggregate all available data to identify rare but functionally important alterations in protein‐coding regions and perturbed molecular pathways that may be therapeutically targetable (Figure 1). This included collating studies of Sanger sequencing hot spot regions in *BRAF*, *NRAS*, and *KIT*, identifying rare but recurrent functionally relevant non‐hot spot variants, which otherwise would not have been uncovered, highlighting the importance of analyzing larger tumor collections.

Acral skin is an extension of the cutaneous surface; however, AM tumors genomically differ to CM, as highlighted in this study and others (Hayward et al., 2017). UVR signatures are present in smaller portions of AMs than that of CM (Hayward et al., 2017), and other risk factors are yet to be elucidated, but trauma and weight‐bearing sites have been proposed as risk factors (Costello et al., 2017; Feibleman et al., 1980; Minagawa et al., 2016; Phan et al., 2006). CMs are genomically classified by the presence of driver mutations in *BRAF*, *NRAS*, and *NF1*, which collectively occur in ~90% of cases (Cancer Genome Atlas, 2015; Hayward et al., 2017), in contrast to ~40% in AM. The major clinical implication centers on the differences in *BRAF* p.V600E frequency, for which vemurafenib‐targeted therapy is standard of care. With less than 1/5 of AMs harboring this *BRAF* mutation, only a small proportion may benefit from this treatment.

The discovery of recurrent *KIT* alterations in AM (Ashida et al., 2009; Beadling et al., 2008; Curtin et al., 2006; Torres‐Cabala et al., 2009) highlighted an ideal target for treatment; however, to date, no approach has improved overall survival (e.g., sunitinib, NCT00577382, Buchbinder et al., 2015; imatinib NCT00424515, Hodi et al., 2013).

While checkpoint inhibitors (anti‐CTLA‐4, PD‐1 or PD‐L1) are highly effective in metastatic CM (Hodi et al., 2010; Larkin et al., 2015; Robert et al., 2011; Wolchok et al., 2013), they are of limited benefit in rare subtypes (Klemen et al., 2020). A retrospective single institutional analysis of anti‐CTLA‐4, PD‐1, or PD‐L1 therapy in patients showed that patients with metastatic CM had median overall survival of 45 months, compared with 17 months for AM (*p* = 0.047), 18 months for MM (*p* = 0.003), and 12 months for UM (*p* < 0.001) (Klemen et al., 2020). Immunotherapy response is associated with a high number of protein altering mutations (Goodman et al., 2017), but this is significantly lower in AM than in CM (Furney et al., 2014; Newell et al., 2020), which likely contributes to lower immunotherapy efficacy. There is, however, immune infiltrate to AM (Castaneda et al., 2019), and alternative methods of immunotherapy might still prove efficacious.

There are several trials in AM currently listed in ClinicalTrials.gov including the following: (a) the PIANO trial (NCT02071940) for efficacy testing of pexidartinib (PLX3397), an inhibitor of CSF‐1R, c‐KIT, and FLT3; (b) PD‐1 antibody (SHR‐1210) in combination with apatinib, an inhibitor of VEGFR‐2 (NCT03955354); and (c) a biomarker study (NCT02978443) of combined nivolumab (PD‐1) and ipilimumab (CTLA‐4) to determine favorable molecular features associated with response rate. It is hoped that with a clearer picture of the genomic alterations present in AM, more targeted clinical trials can be initiated for the largely intractable metastatic disease. Current and recent clinical trials of rare melanoma subtypes were recently reviewed (Alicea & Rebecca, 2020).

This meta‐analysis has strived to identify altered genes and pathways from the conglomeration of published studies, and with a clear picture of the genomic alterations in AM, research needs to focus on the transcriptomic, epigenetic, and proteomic aspects, in particular, how identified aberrations contribute to protein expression and the implications of that on protein pathways, which particularly require considering when selecting therapeutic candidates. Furthermore, the tissue‐specific impact of these variants is an important consideration (Schneider et al., 2017), particularly given the differences in driver events between CM and AM.

In conclusion, this meta‐analysis has comprehensively characterized the genomic alterations in AM; now, these changes need to be taken forward into functional and pharmacological studies to elucidate and test targetable molecular pathways.

## CONFLICT OF INTEREST

The authors declare no potential conflicts of interest.

## Supporting information

Fig S1‐S5

Table S1

Table S2

Supplementary Material

## Data Availability

The data that supports the findings of this study are available in the supplementary material of this article.
