# Supplementary material for: Systematic review and meta‐analysis of genomic alterations in acral melanoma
Source: Pigment Cell Melanoma Res. 2022 Mar 7;35(3):369–86. doi: 10.1111/pcmr.13034 (PMC9540316; doi:10.1111/pcmr.13034)

Supplementary Figures & Legends

Systematic review and meta-analysis of genomic alterations in acral melanoma

Natasa Broit[1,2], Peter A. Johansson[1], Chloe B. Rodgers,[3] Sebastian T. Walpole,[1] Nicholas K. Hayward[1], Antonia L. Pritchard[1, 3]

***

Supplementary Figure Legends

Supplementary Fig. S1 | Further mutations in genes encoding components of cytoplasmic signaling (multiple pathways). A. Mutations in genes encoding components of cytoplasmic signaling (*n* = 181). **B.** Mutations and copy number alterations in genes encoding components of cytoplasmic signaling (*n* = 125).

Supplementary Fig. S2 | Further mutations in genes encoding ligands and receptors (multiple families). A. Mutations in genes encoding ligands and receptors (*n* = 181). **B.** Mutations and copy number alterations in genes encoding ligands and receptors (*n* = 125).

Supplementary Fig. S3 | Further mutations in genes encoding components of the cell cycle. A. Mutations in genes encoding components of the cell cycle (*n* = 181). **B.** Mutations and copy number alterations in genes encoding components of the cell cycle (*n* = 125).

Supplementary Fig. S4 | Further mutations in genes encoding components of DNA repair pathways. A. Mutations in genes encoding proteins involved in DNA repair pathways (*n* = 181). **B.** Mutations and copy number alterations in genes involved in DNA repair pathways (*n* = 125).

Supplementary Fig. S5 | Further mutations in genes encoding proteins involved in chromatin modifications. A. Mutations in genes encoding proteins involved in chromatin modification (*n* = 181). **B.** Mutations and copy number alterations in genes involved in chromatin modifications (*n* = 125).

Supplementary Figure S1

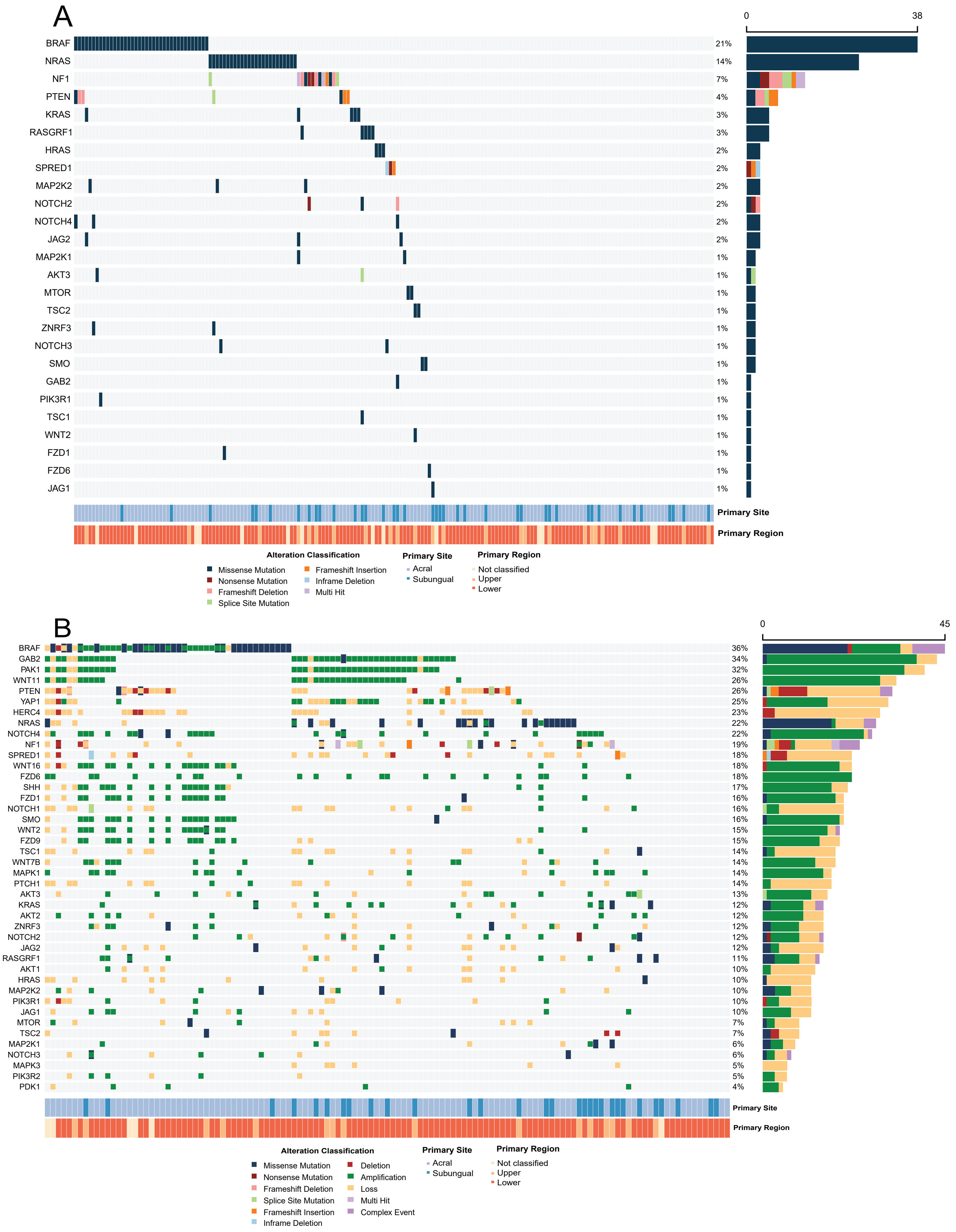

Supplementary Figure S2

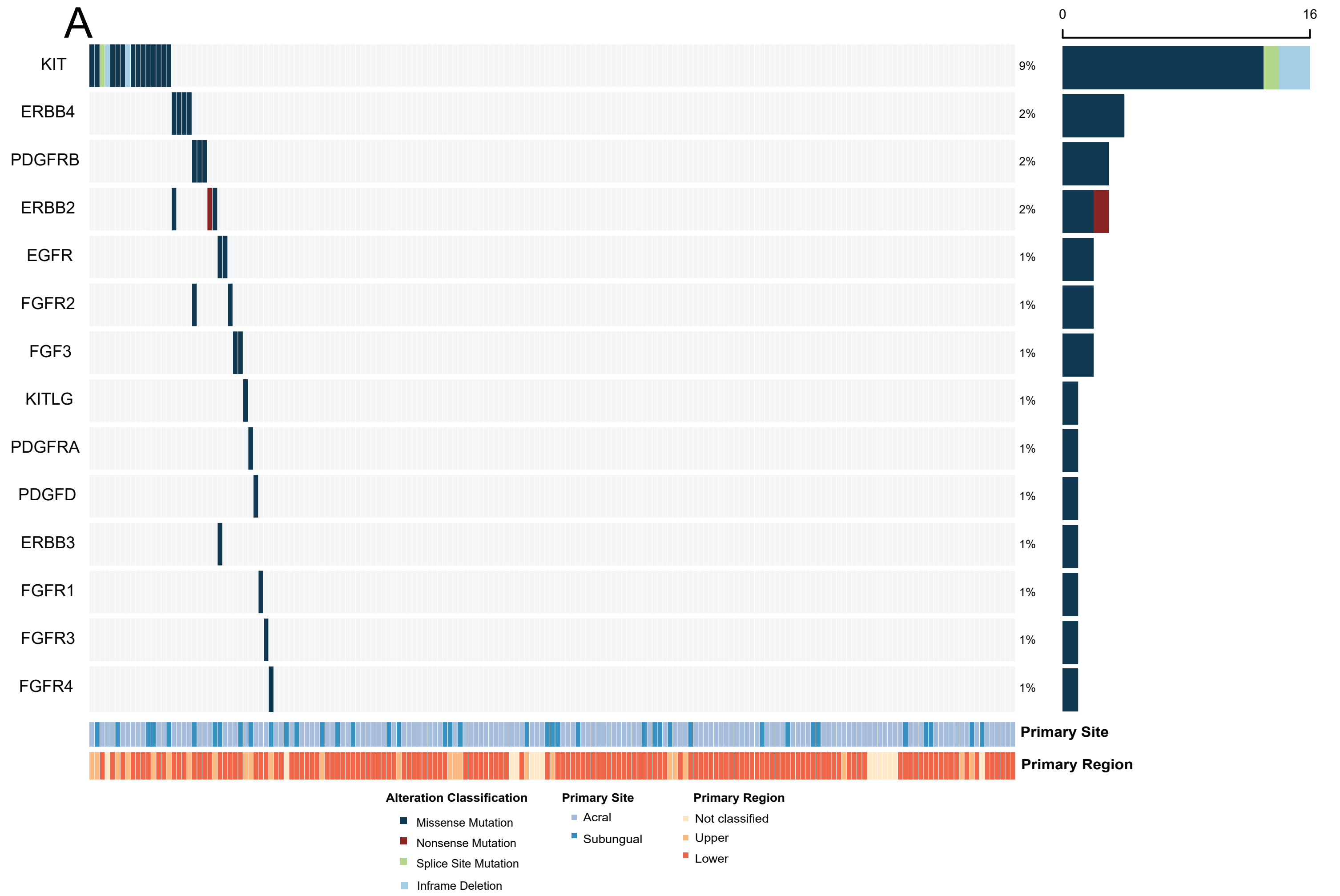

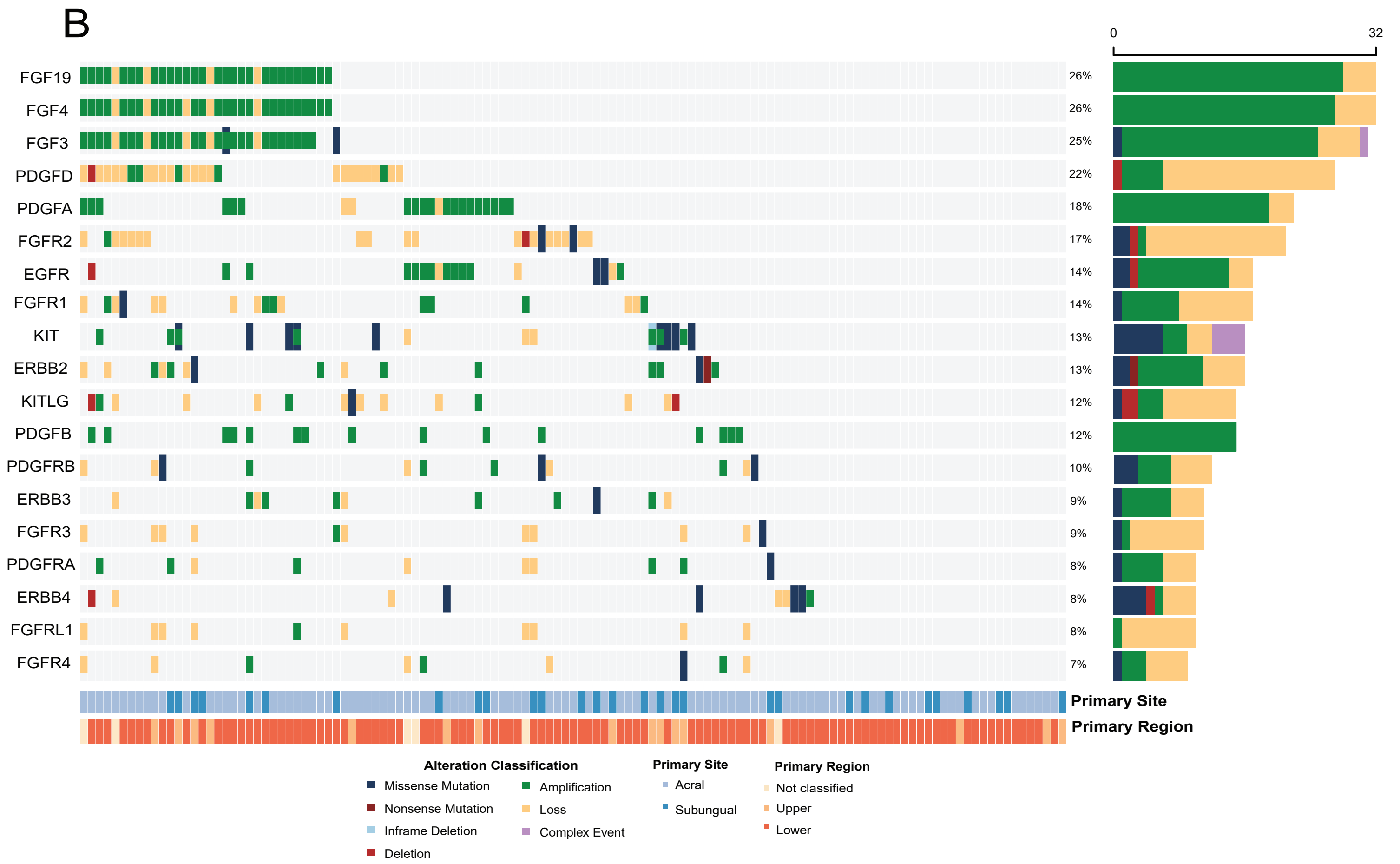

Supplementary Figure S3

A

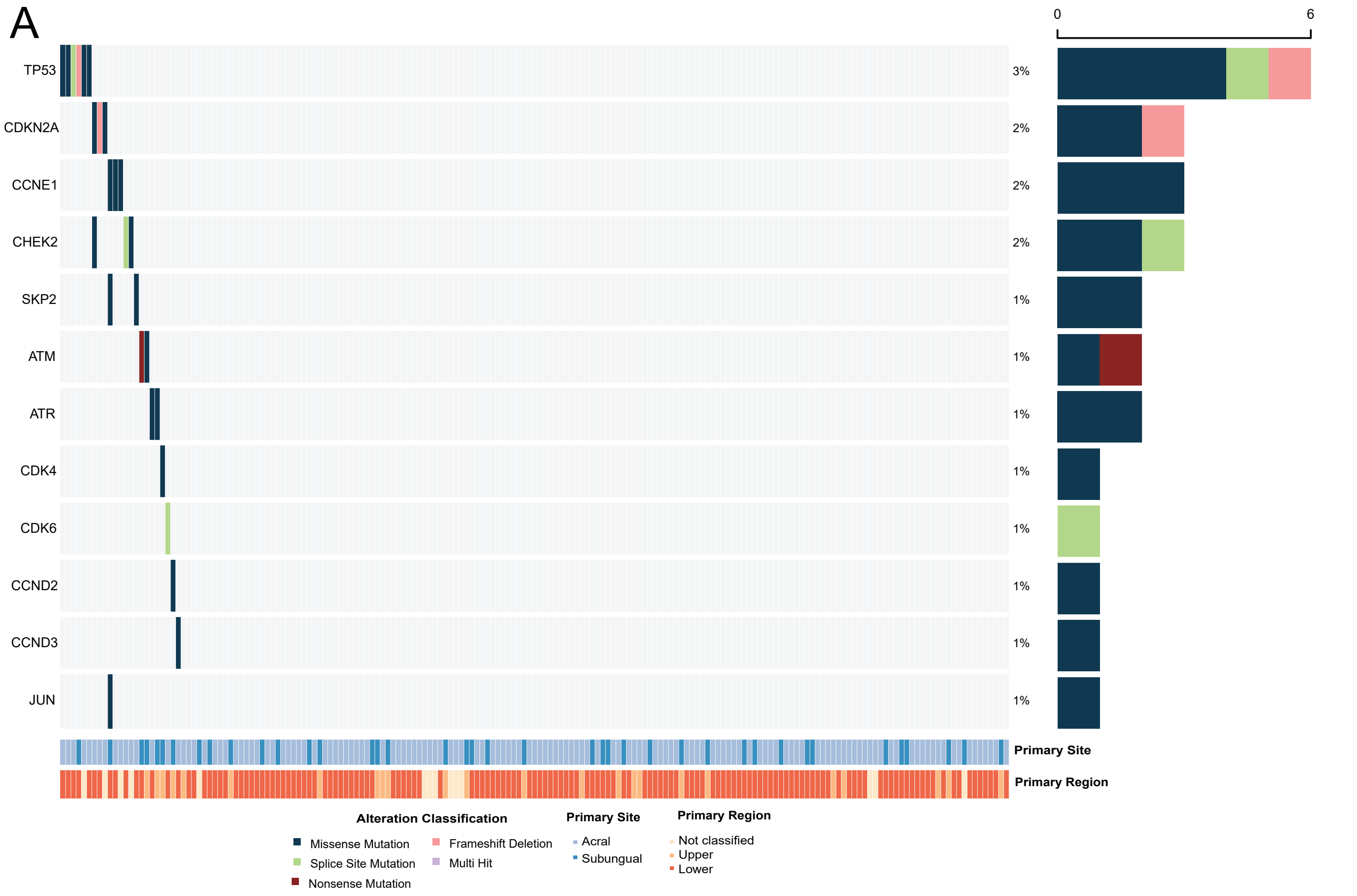

B

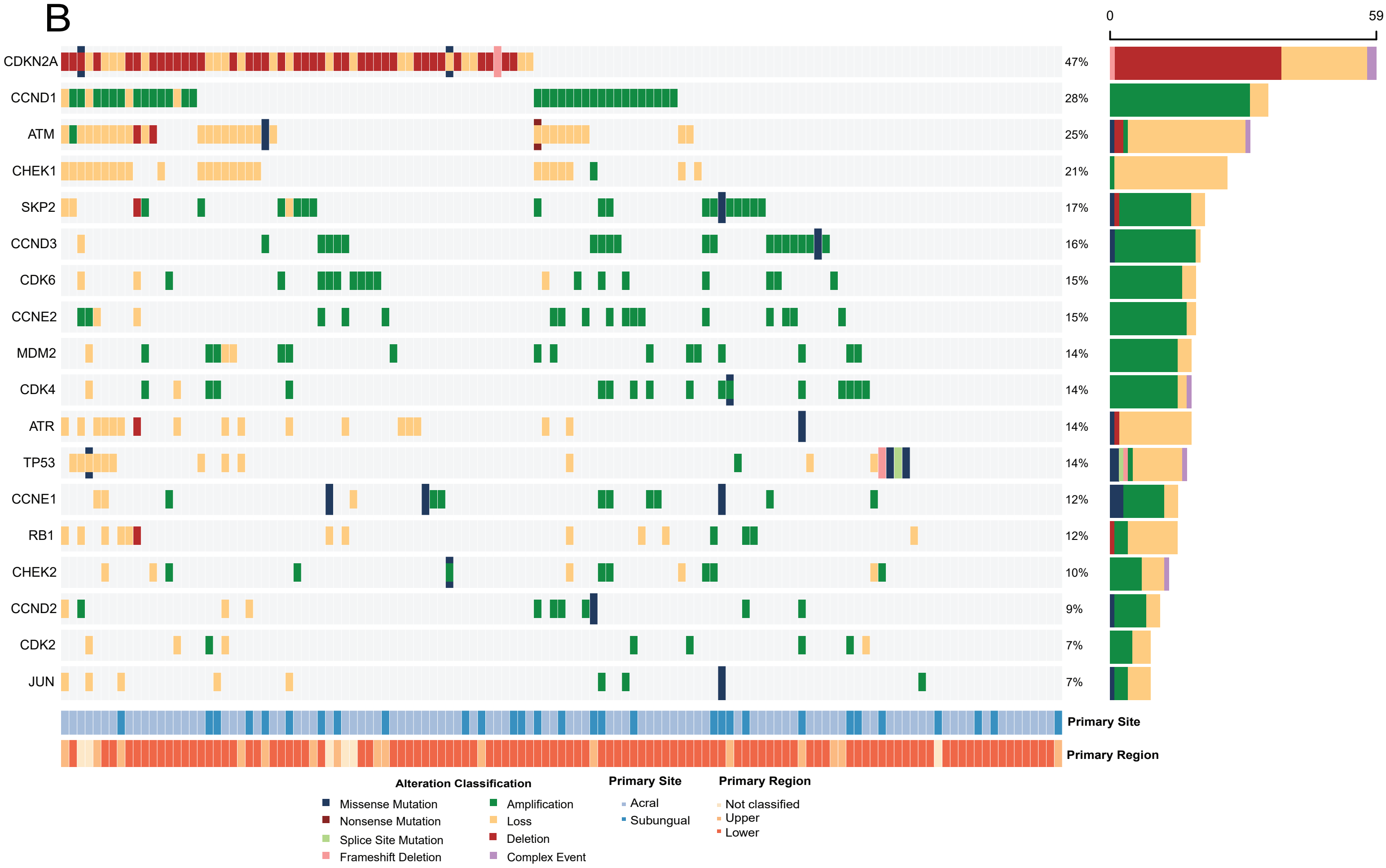

Supplementary Figure S4

A

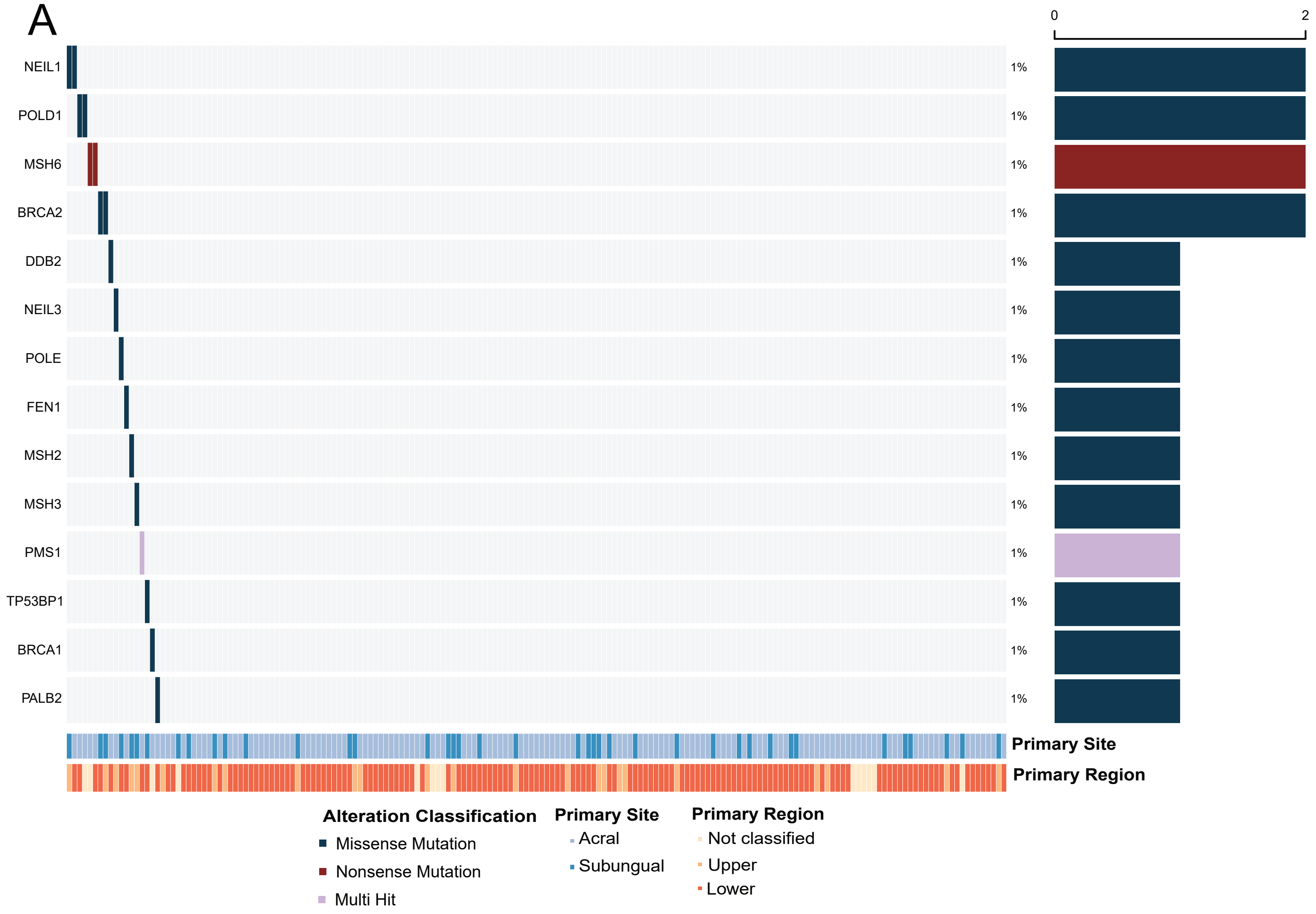

B

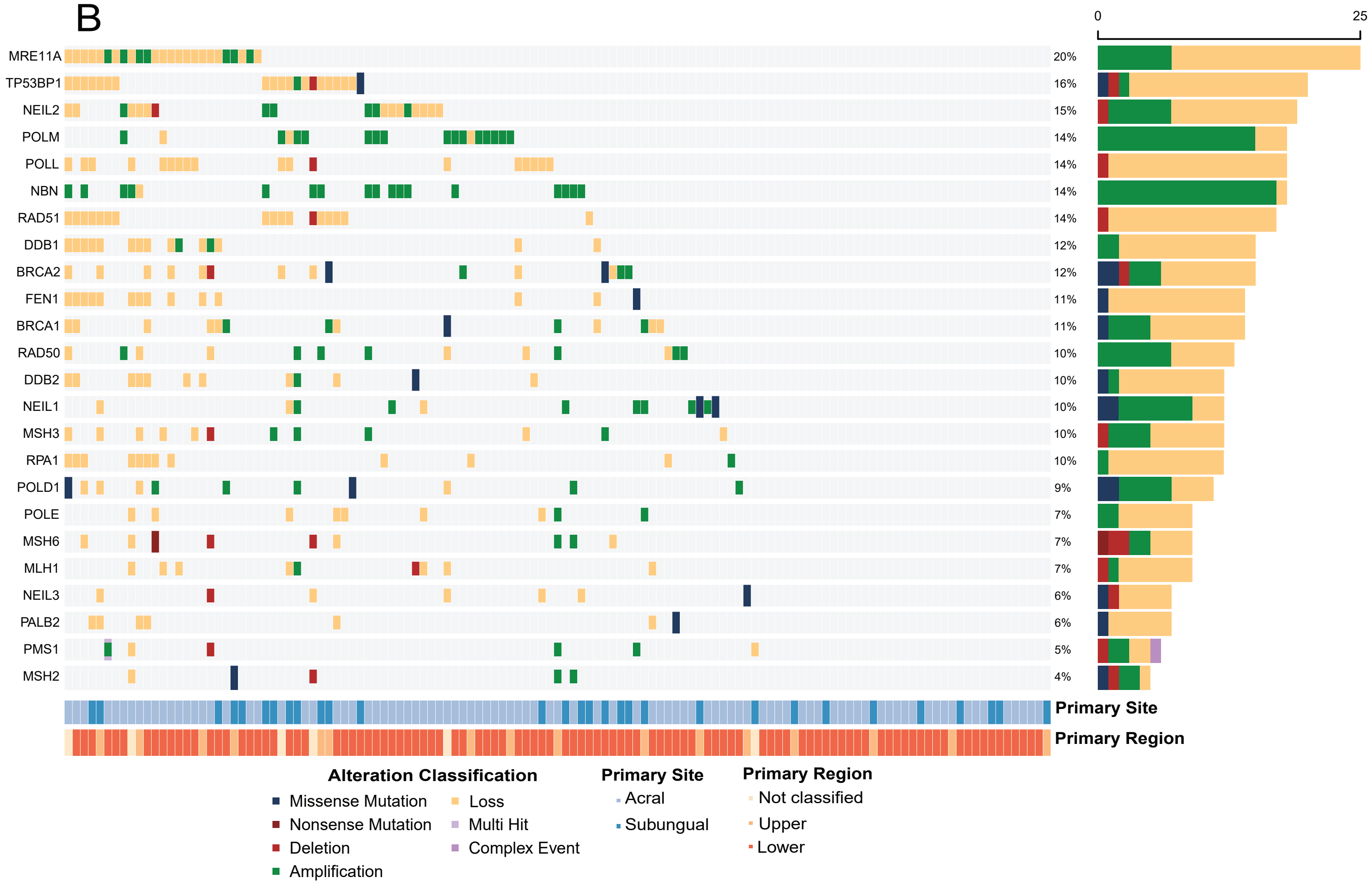

Supplementary Figure S5

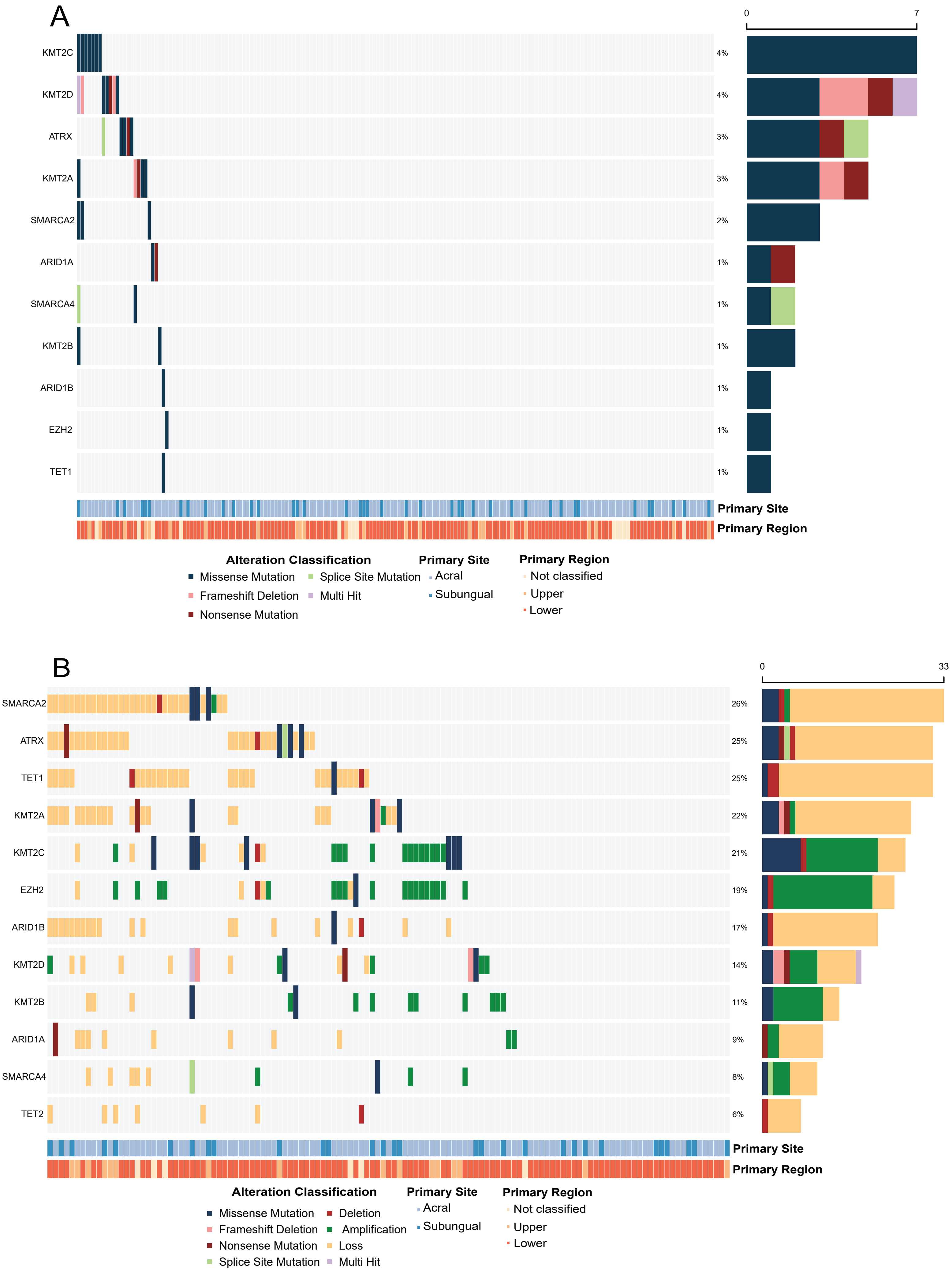

Supplement: Supplementary file 1 — Fig S1‐S5 [file PCMR-35-369-s004.pdf]
