# Supplementary material for: Systematic review and meta‐analysis of genomic alterations in acral melanoma
Source: Pigment Cell Melanoma Res. 2022 Mar 7;35(3):369–86. doi: 10.1111/pcmr.13034 (PMC9540316; doi:10.1111/pcmr.13034)
Supplement: Supplementary file 3 — Table S2 [file PCMR-35-369-s002.pdf]

Table S2A | *BRAF* targeted hotspot studies

Reference	Sub-Type	Exon	Mutation	Mutations (*n*)	Samples (*n*)	Exons Sequenced
(Shim et al., 2017)	Acral & Subungual	15	V600E	2	28	Hotspots of 11, 15
(Choi, Chun, Jin, Lee, & Yun, 2013)	Acral & Subungual	15	V600E	2	33	15
(Zebary et al., 2013)	Acral & Subungual	11	G469A	1	88	11, 15
		15	V600E	13		
		15	V600K	1		
Si, et al	Acral	11	G442S	1	148	11, 15
		11	W450stop	1		
		11	G466R	1		
		15	D594G	1		
		15	V600E	18		
		15	W604stop	1		
(Colombino et al., 2013)	Acral	15	V600E	2	11	15
(Jin et al., 2013)	Acral	15	V600E	3	110	11, 15
(Oyama et al., 2015)	Acral	15	V600E	13	20	15
(Lang & MacKie, 2005)	Acral	15	V600E	2	13	15
(Puig-Butille et al., 2013)	Acral	None	None	None	17	11, 15
(X. Kang et al., 2018)	Acral	15	L597Q	1	19	11, 15
		11	P453H	1		
(Moon et al., 2018)	Acral	15	V600E	17	64	1 - 18
		11	Y471_K472insYGTVY	1		
		15	K601N	1		
		15	D594G	1		
		15	G596R	1		
		1	D22N	1		
(Ashida, Takata, Murata, Kido, & Saida, 2009)	Acral	15	V600E	3	22	15
(Akslen et al., 2008)	Acral	11	G466V	1	24	11, 15
		15	V600E	1	25	
	Acral	15	V600E	18	84	15

(Carvajal et al., 2011)		15	I592del	1		
(Dika et al., 2020)	Acral & Subungual	15	V600E	3	31	15
		15	K601E	1		
(Gao, Tsai, Perng, Wang, & Chiang, 2018)	Acral	15	V600E	6	40	11, 15
		15	V600L	6		
(Yun et al., 2011)	Acral	/	None	/	49	15
(Zaremba et al., 2019)	Acral & Subungual	15	V600E	15	50	1 - 18
(Shi et al., 2019)	Acral	15	V600E	6	29	1 - 18
		15	V600K	1		
		15	K601E	1		
(Yeh et al., 2019)	Acral	15	V600E	21	122	1 -18
		11	G469S	1		
		15	K601E	1		
		15	V600K	3		
(Hilke et al., 2020)	Acral	15	V600E	1	14	1 - 18
(Borkowska et al., 2020)	Subungual	15	V600E	1	31	Hotspots of 11, 15
(Zou et al., 2020)	Acral	15	V600E	10	54	1 - 18
		15	V600E, T599R	1		
		15	L597Q, D565E	1		
		11	G466A	1		
		15, 10	V600E, S394P	1		
		11	G469A	1		
(Cirenajwis et al., 2017)	Acral	15	V600E	1	6	1 - 18
(Sheen et al., 2020)	Acral	15	V600E	3	45	1 - 18
		12	E501K	1		
(Niu et al., 2013)	Acral	15	V600E	5	30	Hotspots of 11, 15

Table S2B | *NRAS* targeted hotspot studies

Reference	Sub-Type	Exon	Mutation	Mutations (*n*)	Samples (*n*)	Exons Sequenced
(Uhara et al., 2014)	Acral	3	Q61K	4	54	2, 3
		2	G12A	1		
(Zebary et al., 2013)	Acral & Subungual	2	G12C	1	88	2, 3
		2	D33E	1		
		3	Q61R	8		
		3	Q61L	2		
		3	Q61K	1		
(Si et al., 2012)	Acral	2	G12C	1	148	2, 3
		2	G13R	1		
		3	A59D	1		
		3	Q61K	1		
		3	Q61L	1		
		3	Q61R	8		
(Oyama et al., 2015)	Acral	None	None	None	20	2
(Puig-Butille et al., 2013)	Acral	2	G12V	2	17	2, 3
		3	Q61H	1		
(Moon et al., 2018)	Acral	3	Q61R	4	64	1 - 7
		2	G12D	2		
		3	S173R	1		
		2	G13R	2		
		3	Q61K	3		
		3	Y64D	1		
		3	Q61H	1		
(Ashida et al., 2009)	Acral	None	None	None	22	3
(Sheen et al., 2016)	Acral	2	G13D	2	89	2, 3
		3	Q61L	6		
		3	Q61R	1		
(Akslen et al., 2008)	Acral	2	G12D	3	26	2, 3
		3	None	0	24	

(Carvajal et al., 2011)	Acral	3	T58I, Y64H	1	84	2, 3
		3	Q61K	4		
		3	Q61H	2		
		3	Q61R	1		
		2	G12C	3		
		2	G12V	1		
		2	G13R	1		
(Dika et al., 2020)	Acral Melanoma and Subungual	2	G13R	1	30	2, 3
		3	Q61R	1		
		3	Q61R & Q43stop	1		
		3	L79F & C80Y	1		
		3	E62K	1		
		2	G12V	1		
(Gao et al., 2018)	Acral Melanoma	2	G13R	2	40	2, 3
		2	G12D	2		
(Zaremba et al., 2019)	Acral and Subungual	3	Q61K	3	50	1 - 7
		5	A155V	1		
		3	Q61R	6		
		3	Q61H	1		
		2	G12S	1		
		2	G12D	2		
(Shi et al., 2019)	Acral	2	G13R	1	29	1 - 7
		3	Q61R	2		
(Yeh et al., 2019)	Acral	2	G12A	1	122	1 - 7
		2	G12C	3		
		2	G13D	1		
		2	G13R	3		
		3	Q61H	2		
		3	Q61K	7		
		3	Q61R	17		
(Hilke et al., 2020)	Acral	3	Q61R	1	14	1 - 7
	Subungual	2	G12C	1	31	Hotspots of 2, 3

(Borkowska et al., 2020)		2	G13D	1		
		3	Q61R	1		
		3	Q61K	1		
(Zou et al., 2020)	Acral	2	G12C	1	54	1 - 7
		2	G13D	1		
		3	Q61K	3		
(Cirenajwis et al., 2017)	Acral	3	Q61R	1	6	1 - 7
(Sheen et al., 2020)	Acral	3	Q61L	1	45	1 - 7
		3	Q61K	2		
		4	A146T	1		
		2	G13D	1		
		2	G12D	1		
(Niu et al., 2013)	Acral	2	G12C	1	30	Hotspots of 2, 3
		3	Q61L	1		

Table S2C | *KIT* targeted hotspot studies

Reference	Sub-Type	Exon	Mutation	Mutations (*n*)	Samples (*n*)	Exons Sequenced
(Torres-Cabala et al., 2009)	Acral	11	L576P	1	39	11, 13, 17
		17	D816V	1		
		11	N566-D572 del	1		
		13	N655K	1		
		11	V559A	1		
(Abu-Abed et al., 2012)	Acral	13	K642E	1	24	11, 13
(Curtin, Busam, Pinkel, & Bastian, 2006)	Acral	11	Y553N	1	28	11, 13, 17, 18
		13	R634W	1		
		13	K642E	1		
(Shim et al., 2017)	Acral & Subungual	11	L576P	2	28	Hotspots of 11, 13
		13	V654A	1		
(Dai et al., 2013)	Acral & Subungual	9	A502V	1	39	9, 11, 13, 17
		11	E583_F584insPYDHKWE	1		
		9	A507T	1		
		13	V654M	1		
		9	V474I	1		
		9	V489I & A493T	1		
		17	G803D	1		
		13	K642E	1		
		17	D816V	1		
(Kong et al., 2011)	Acral	9	V489I	1	193	9, 11, 13, 17, 18
		9	E490G	1		
		11	Y553N	1		
		11	I563V	1		
		11	W582stop	1		
		11	N566D & A829V	1		
		11	L576P	6		
		11	L576F	1		
		13	E633G	1		
		13	G648S	1		
		13	K642E	1		
		17	I817T	1		
		17	N822K	1		

		18	C844Y	1		
		18	W853stop	1		
		18	L859P	1		
(Zebary et al., 2013)	Acral & Subungual	11	L576P	5	88	9, 11, 13, 17, 18
		11	V560D	1		
		11	D572G	1		
		11	V559del	1		
		11	D579del	2		
		11	P577del	1		
		13	K642E	1		
		17	Y823C	1		
(X. J. Kang et al., 2016)	Acral	13	K642E	1	35	11, 13
		11	L576P	5		
(Colombino et al., 2013)	Acral	11	D579del	1	11	9, 11, 13, 17, 18
(Jin et al., 2013)	Acral	9	V474I	1	110	9, 11, 13, 17
		11	L576P	2		
		13	S639P	1		
		13	K642E	2		
		13	G664S	1		
		11, 17	L576P & I817L	1		
(Oyama et al., 2015)	Acral	9	V489G	1	20	9, 11, 13, 17, 18
		11	R588K	1		
		11	D579G	1		
(Moon et al., 2018)	Acral	1	R19H & V654A	1	64	1 - 21
		11	V560D	1		
		17, 11	I817L & L576P	1		
		17	Y823D	1		
		13	K642E	2		
		11	L576P	1		
(Lin et al., 2013)	Acral	none	none	None	20	11, 13, 17, 18
(Ashida et al., 2009)	Acral	13	K642E	1	16	11, 13, 17, 18
		17	D820Y	1		
(Schoenewolf et al., 2012)	Acral	11	L576P	2	41	9, 11, 13, 17, 18
		13	K642E	1		
		13	T632I	1		

(Handolias et al., 2010)	Acral	17	D820Y	1	16	11, 13, 17
(Puntervoll, Molven, & Akslen, 2014)	Acral<br>Acral	11	D579del	1	36	9, 11, 13, 17, 18
		18	S864L	1		
		13	K642E	1		
(Carvajal et al., 2011)	Acral	11	L576P	3	84	9, 11, 13, 17, 18
		11	V559G	1		
		11	W557R	1		
		13	K642E	3		
		13	I653T	1		
		13	G648D	1		
		17	D820Y	1		
		17	L813P	1		
		18	P838L	1		
		18	S850G	1		
		11, 18	E554K, Y846C	1		
		17	K818Q, N822Y	1		
		13	L647F, G648D	1		
		18	S840I, L859P, L862P	1		
(Comodo-Navarro et al., 2020)	Acral	11	D572N	1	25	11, 13
		11, 13	P585T & Y646H	1		
		11	P585T & Y568D	1		
		11	Y578C	1		
		13	K642R	2		
		13	G658E	1		
		11	P585T & M552I	1		
		13	C660R	1		
(Dika et al., 2020)	Acral and Subungual	13	N655K	1	29	9, 11, 13, 17
		13	K642E	1		
		11	P551L	1		
		17	D816N	1		
		11	V560D	1		
(Terada, 2010)	Acral	11	V559A	1	2	9, 11, 13, 17
(Yun et al., 2011)	Acral	11	K558R	1	92	11, 13, 17, 18
		11	V559A	1		
		11	L576P	1		

		13	N655K	1		
(Minor et al., 2012)	Acral	13	E635G	1	22	9, 11, 13, 17, 18
		11	W557R, G565E	1		
		11	L576P	1		
(Zaremba et al., 2019)	Acral and Subungual	11	L576P	1	50	1 - 21
		13	K642E	1		
		18	A829P	1		
(Shi et al., 2019)	Acral	11	L576P	1	29	1 - 21
		11	Y578C	1		
		17	D820Y	1		
		16	E761stop	1		
(Yeh et al., 2019)	Acral	17	D816V	1	122	1 - 21
		17	D820G	1		
		17	D820Y	1		
		13	K642E	3		
		11	L576P	4		
		17	N822K	1		
		11	T574delinsTQLPYDQT	1		
		11	V559D	1		
		11	W557R	1		
(Hilke et al., 2020)	Acral	13	K642E	1	14	1 - 21
		13	S628G	1		
		8	C443S	1		
(Borkowska et al., 2020)	Subungual	11	Gln575_Pro577del	1	31	Hotspots of 11, 13
		11	L576P	1		
		11	L576P, V559D	1		
		11	Asp579del	1		
(Zou et al., 2020)	Acral	17	D816H	1	54	1 - 21
		11	V559D	1		
		11	P573_D579dup	1		
		17	S840N, D820V	1		
		11	L576P	2		
		17	A829P	1		
		13	K642E	1		
(Cirenajwis et al., 2017)	Acral	13	K642E	1	6	1 - 21
		17	N822K	1		

(Sheen et al., 2020)	Acral	17	N822K	2	45	1 - 21
		13	K642E	2		
		17	D820Y	1		
		3	T84M	1		
		11	L576P	4		
		11	D572G	1		
(Niu et al., 2013)	Acral	13	K642E	1	30	Hotspots of 11, 13
		11	L576P	1		

References

Abu-Abed, S., Pennell, N., Petrella, T., Wright, F., Seth, A., & Hanna, W. (2012). KIT gene mutations and patterns of protein expression in mucosal and acral melanoma. *J Cutan Med Surg, 16*(2), 135-142. doi:10.2310/7750.2011.11064

Akslen, L. A., Puntervoll, H., Bachmann, I. M., Straume, O., Vuhahula, E., Kumar, R., & Molven, A. (2008). Mutation analysis of the EGFR-NRAS-BRAF pathway in melanomas from black Africans and other subgroups of cutaneous melanoma. *Melanoma Res, 18*(1), 29-35. doi:10.1097/CMR.0b013e3282f32517

Ashida, A., Takata, M., Murata, H., Kido, K., & Saida, T. (2009). Pathological activation of KIT in metastatic tumors of acral and mucosal melanomas. *Int J Cancer, 124*(4), 862-868. doi:10.1002/ijc.24048

Borkowska, A., Szumera-Cieckiewicz, A., Spalek, M., Teterycz, P., Czarnecka, A., Kowalik, A., & Rutkowski, P. (2020). Mutation profile of primary subungual melanomas in Caucasians. *Oncotarget, 11*(25), 2404-2413. doi:10.18632/oncotarget.27642

Carvajal, R. D., Antonescu, C. R., Wolchok, J. D., Chapman, P. B., Roman, R. A., Teitcher, J., . . . Schwartz, G. K. (2011). KIT as a therapeutic target in metastatic melanoma. *JAMA, 305*(22), 2327-2334. doi:10.1001/jama.2011.746

Choi, Y. D., Chun, S. M., Jin, S. A., Lee, J. B., & Yun, S. J. (2013). Amelanotic acral melanomas: clinicopathological, BRAF mutation, and KIT aberration analyses. *J Am Acad Dermatol, 69*(5), 700-707. doi:10.1016/j.jaad.2013.06.035

Cirenajwis, H., Lauss, M., Ekedahl, H., Torngren, T., Kvist, A., Saal, L. H., . . . Jonsson, G. (2017). NF1-mutated melanoma tumors harbor distinct clinical and biological characteristics. *Mol Oncol, 11*(4), 438-451. doi:10.1002/1878-0261.12050

Colombino, M., Lissia, A., Franco, R., Botti, G., Ascierto, P. A., Manca, A., . . . Cossu, A. (2013). Unexpected distribution of cKIT and BRAF mutations among southern Italian patients with sinonasal melanoma. *Dermatology, 226*(3), 279-284. doi:10.1159/000350683

Comodo-Navarro, A. N., Fernandes, M., Barcelos, D., Carapeto, F. C. L., Guimaraes, D. P., de Sousa Moraes, L., . . . Landman, G. (2020). Intratumor Heterogeneity of KIT Gene Mutations in Acral Lentiginous Melanoma. *Am J Dermatopathol, 42*(4), 265-271. doi:10.1097/DAD.0000000000001475

Curtin, J. A., Busam, K., Pinkel, D., & Bastian, B. C. (2006). Somatic activation of KIT in distinct subtypes of melanoma. *J Clin Oncol, 24*(26), 4340-4346. doi:10.1200/JCO.2006.06.2984

Dai, B., Cai, X., Kong, Y. Y., Yang, F., Shen, X. X., Wang, L. W., & Kong, J. C. (2013). Analysis of KIT expression and gene mutation in human acral melanoma: with a comparison between primary tumors and corresponding metastases/recurrences. *Hum Pathol, 44*(8), 1472-1478. doi:10.1016/j.humpath.2013.01.007

Dika, E., Veronesi, G., Altimari, A., Riefolo, M., Ravaioli, G. M., Piraccini, B. M., . . . Patrizi, A. (2020). BRAF, KIT, and NRAS Mutations of Acral Melanoma in White Patients. *Am J Clin Pathol, 153*(5), 664-671. doi:10.1093/ajcp/aqz209

Gao, H. W., Tsai, W. C., Perng, C. L., Wang, W. M., & Chiang, C. P. (2018). Distinct MAPK and PI3K pathway mutations in different melanoma types in Taiwanese individuals. *Eur J Dermatol, 28*(4), 509-518. doi:10.1684/ejd.2018.3359

Handolias, D., Hamilton, A. L., Salemi, R., Tan, A., Moodie, K., Kerr, L., . . . McArthur, G. A. (2010). Clinical responses observed with imatinib or sorafenib in melanoma patients expressing mutations in KIT. *Br J Cancer, 102*(8), 1219-1223. doi:10.1038/sj.bjc.6605635

Hilke, F. J., Sinnberg, T., Gschwind, A., Niessner, H., Demidov, G., Amaral, T., . . . Forschner, A. (2020). Distinct Mutation Patterns Reveal Melanoma Subtypes and Influence Immunotherapy Response in Advanced Melanoma Patients. *Cancers (Basel), 12*(9). doi:10.3390/cancers12092359

Jin, S. A., Chun, S. M., Choi, Y. D., Kweon, S. S., Jung, S. T., Shim, H. J., & Yun, S. J. (2013). BRAF mutations and KIT aberrations and their clinicopathological correlation in 202 Korean melanomas. *J Invest Dermatol, 133*(2), 579-582. doi:10.1038/jid.2012.338

Kang, X., Zeng, Y., Liang, J., Li, J., Ren, D., Chai, L., . . . Wang, W. (2018). Aberrations and clinical significance of BRAF in malignant melanoma: A series of 60 cases in Chinese Uyghur. *Medicine (Baltimore), 97*(1), e9509. doi:10.1097/MD.0000000000009509

Kang, X. J., Shi, X. H., Chen, W. J., Pu, X. M., Sun, Z. Z., Halifu, Y., . . . Ren, D. Y. (2016). Analysis of KIT mutations and c-KIT expression in Chinese Uyghur and Han patients with melanoma. *Clin Exp Dermatol, 41*(1), 81-87. doi:10.1111/ced.12659

Kong, Y., Si, L., Zhu, Y., Xu, X., Corless, C. L., Flaherty, K. T., . . . Guo, J. (2011). Large-scale analysis of KIT aberrations in Chinese patients with melanoma. *Clin Cancer Res, 17*(7), 1684-1691. doi:10.1158/1078-0432.CCR-10-2346

Lang, J., & MacKie, R. M. (2005). Prevalence of exon 15 BRAF mutations in primary melanoma of the superficial spreading, nodular, acral, and lentigo maligna subtypes. *J Invest Dermatol, 125*(3), 575-579. doi:10.1111/j.0022-202X.2005.23833.x

Lin, Y. C., Chang, Y. M., Ho, J. Y., Lin, H. C., Tsai, Y. M., Chiang, C. P., . . . Gao, H. W. (2013). C-kit expression of melanocytic neoplasm and association with clinicopathological parameters and anatomic locations in Chinese people. *Am J Dermatopathol, 35*(5), 569-575. doi:10.1097/DAD.0b013e318279566a

Minor, D. R., Kashani-Sabet, M., Garrido, M., O'Day, S. J., Hamid, O., & Bastian, B. C. (2012). Sunitinib therapy for melanoma patients with KIT mutations. *Clin Cancer Res, 18*(5), 1457-1463. doi:10.1158/1078-0432.CCR-11-1987

Moon, K. R., Choi, Y. D., Kim, J. M., Jin, S., Shin, M. H., Shim, H. J., . . . Yun, S. J. (2018). Genetic Alterations in Primary Acral Melanoma and Acral Melanocytic Nevus in Korea: Common Mutated Genes Show Distinct Cytomorphological Features. *J Invest Dermatol, 138*(4), 933-945. doi:10.1016/j.jid.2017.11.017

Niu, H. T., Zhou, Q. M., Wang, F., Shao, Q., Guan, Y. X., Wen, X. Z., . . . Zhang, X. S. (2013). Identification of anaplastic lymphoma kinase break points and oncogenic mutation profiles in acral/mucosal melanomas. *Pigment Cell Melanoma Res, 26*(5), 646-653. doi:10.1111/pcmr.12129

Oyama, S., Funasaka, Y., Watanabe, A., Takizawa, T., Kawana, S., & Saeki, H. (2015). BRAF, KIT and NRAS mutations and expression of c-KIT, phosphorylated extracellular signal-regulated kinase and phosphorylated AKT in Japanese melanoma patients. *J Dermatol, 42*(5), 477-484. doi:10.1111/1346-8138.12822

Puig-Butille, J. A., Badenas, C., Ogbah, Z., Carrera, C., Aguilera, P., Malvehy, J., & Puig, S. (2013). Genetic alterations in RAS-regulated pathway in acral lentiginous melanoma. *Exp Dermatol, 22*(2), 148-150. doi:10.1111/exd.12080

Puntervoll, H. E., Molven, A., & Akslen, L. A. (2014). Frequencies of KIT and GNAQ mutations in acral melanoma. *J Cutan Pathol, 41*(11), 893-894. doi:10.1111/cup.12382

Schoenewolf, N. L., Bull, C., Belloni, B., Holzmann, D., Tonolla, S., Lang, R., . . . Dummer, R. (2012). Sinonasal, genital and acrolentiginous melanomas show distinct characteristics of KIT expression and mutations. *Eur J Cancer, 48*(12), 1842-1852. doi:10.1016/j.ejca.2012.02.049

Sheen, Y. S., Liao, Y. H., Liau, J. Y., Lin, M. H., Hsieh, Y. C., Jee, S. H., & Chu, C. Y. (2016). Prevalence of BRAF and NRAS mutations in cutaneous melanoma patients in Taiwan. *J Formos Med Assoc, 115*(2), 121-127. doi:10.1016/j.jfma.2015.02.001

Sheen, Y. S., Tan, K. T., Tse, K. P., Liao, Y. H., Lin, M. H., Chen, J. S., . . . Chu, C. Y. (2020). Genetic alterations in primary melanoma in Taiwan. *Br J Dermatol, 182*(5), 1205-1213. doi:10.1111/bjd.18425

Shi, K., Zhang, B., Kong, B. Y., Zhang, Y., Igartua, C., Mohan, L. S., . . . Gerami, P. (2019). Distinct Genomic Features in a Retrospective Cohort of Mucosal, Acral and Vulvovaginal Melanomas. *J Am Acad Dermatol*. doi:10.1016/j.jaad.2019.07.017

Shim, J. H., Shin, H. T., Park, J., Park, J. H., Lee, J. H., Yang, J. M., . . . Lee, D. Y. (2017). Mutational profiling of acral melanomas in Korean populations. *Exp Dermatol, 26*(10), 883-888. doi:10.1111/exd.13321

Si, L., Kong, Y., Xu, X., Flaherty, K. T., Sheng, X., Cui, C., . . . Guo, J. (2012). Prevalence of BRAF V600E mutation in Chinese melanoma patients: large scale analysis of BRAF and NRAS mutations in a 432-case cohort. *Eur J Cancer, 48*(1), 94-100. doi:10.1016/j.ejca.2011.06.056

Terada, T. (2010). Low incidence of KIT gene mutations and no PDGFRA gene mutations in primary cutaneous melanoma: an immunohistochemical and molecular genetic study of Japanese cases. *Int J Clin Oncol, 15*(5), 453-456. doi:10.1007/s10147-010-0087-0

Torres-Cabala, C. A., Wang, W. L., Trent, J., Yang, D., Chen, S., Galbincea, J., . . . Ivan, D. (2009). Correlation between KIT expression and KIT mutation in melanoma: a study of 173 cases with emphasis on the acral-lentiginous/mucosal type. *Mod Pathol, 22*(11), 1446-1456. doi:10.1038/modpathol.2009.116

Uhara, H., Ashida, A., Koga, H., Ogawa, E., Uchiyama, A., Uchiyama, R., . . . Okuyama, R. (2014). NRAS mutations in primary and metastatic melanomas of Japanese patients. *Int J Clin Oncol, 19*(3), 544-548. doi:10.1007/s10147-013-0573-2

Yeh, I., Jorgenson, E., Shen, L., Xu, M., North, J. P., Shain, A. H., . . . Asgari, M. M. (2019). Targeted Genomic Profiling of Acral Melanoma. *J Natl Cancer Inst, 111*(10), 1068-1077. doi:10.1093/jnci/djz005

Yun, J., Lee, J., Jang, J., Lee, E. J., Jang, K. T., Kim, J. H., & Kim, K. M. (2011). KIT amplification and gene mutations in acral/mucosal melanoma in Korea. *APMIS, 119*(6), 330-335. doi:10.1111/j.1600-0463.2011.02737.x

Zaremba, A., Murali, R., Jansen, P., Moller, I., Sucker, A., Paschen, A., . . . Cosgarea, I. (2019). Clinical and genetic analysis of melanomas arising in acral sites. *Eur J Cancer, 119*, 66-76. doi:10.1016/j.ejca.2019.07.008

Zebary, A., Omholt, K., Vassilaki, I., Hoiom, V., Linden, D., Viberg, L., . . . Hansson, J. (2013). KIT, NRAS, BRAF and PTEN mutations in a sample of Swedish patients with acral lentiginous melanoma. *J Dermatol Sci, 72*(3), 284-289. doi:10.1016/j.jdermsci.2013.07.013

Zou, Z., Ou, Q., Ren, Y., Lv, Q., Qin, L., Zhao, L., . . . Liu, B. (2020). Distinct genomic traits of acral and mucosal melanomas revealed by targeted mutational profiling. *Pigment Cell Melanoma Res, 33*(4), 601-611. doi:10.1111/pcmr.12865
