# Supplementary material for: Systematic review and meta‐analysis of genomic alterations in acral melanoma
Source: Pigment Cell Melanoma Res. 2022 Mar 7;35(3):369–86. doi: 10.1111/pcmr.13034 (PMC9540316; doi:10.1111/pcmr.13034)
Supplement: Supplementary file 4 — Supplementary Material [file PCMR-35-369-s001.docx]

**Supplementary Methods**

**Data acquisition**

*Main cohort studies*

The main cohort included WGS and WES FF samples with matched normal tissue (Main Manuscript, **Table 1**). Ten studies were identified in the literature that matched these criteria [1-10]. The data were accessed through various sources.

Six of these studies host their data on cBioPortal (cbioportal.org/) [11, 12] under the study names: “*Acral Melanoma (TGEN, Genome Res, 2017)*” by Liang et al. [2]; “*Skin Cutaneous Melanoma (Yale, Nat Genet, 2012)*” by Krauthammer et al. [8]; “*Skin Cutaneous Melanoma (Broad, Cell, 2012****)***” by Hodis et al. [7]; “*Melanoma (Broad/Dana Farber, Nature, 2012*” by Berger et al. [5]; “*Melanoma (MSKCC, NEJM, 2014)*” by Snyder et al. [9]; “*Skin Cutaneous Melanoma (TCGA, PanCancer Atlas)*” by the Cancer Genome Atlas [10].

Data from four studies were access directly from the Supplementary Tables from each respective manuscript [1, 3, 4, 6].

The data were cross checked using information provided by each study and the sample specific information (e.g. sample name, clinical data, meta-data and genomic profile); for example, the 35 samples reported by Hayward et al. [4] were also published in Newell et al. [1] and were excluded.

Copy number alterations (CNA) were available from Liang et al., 2017 [2] and Newell et al., 2020 [1] as GISTIC reports, allowing the data from these studies to be analysed together.

Tumour mutation burden (TMB) was calculated by tallying non-synonymous mutations in each tumour in each study. Given that TMB could be reliant on individual study factors, such as analysis pipeline and intrinsic tumour features, TMB were compared between studies (Figure I).

**Figure I: Comparison of TMB across the studies, based on non-synonymous tumour calls**.

These data indicate statistically significant differences between the studies, precluding combination to analyse TMB as a total cohort (Figure I). We further investigated if this was driven by differences in proportion of subungual vs. palmar/plantar tumours in each cohort, as the subungual subtype is associated with higher TMB [1]; Figure II. This revealed both subsets were significantly different between the different cohorts, suggesting this was not the underlying cause.

**Figure II: TMB vs. location of AM**. The subungual and acral skin tumours were separated out from each individual study to investigate if there was a bias towards one tumour location compared to another in a given study. The studies were still statistically different to each other, when compared only between AM subtype (acral *P* <0.0001; subungual *P* = 0.013)

Finally, the association between TMB and UVR signatures (SBS7a-d) and TMB in each study was assessed (Figure III). This analysis revealed that within each study, the higher TMB tended to coincide with the presence of mutational signatures associated with UVR (SBS7a-d). This did not, however, reveal any bias within individual studies towards an over/under representation of tumours with higher UVR mutational signatures, barring Krauthammer et al. [8], who did not sequence a tumour with any UVR signature and had notably lower TMB compared to the other eight studies (Figure III).

**Figure III: TMB vs. SBS7a-d in each individual study**. The individual samples within each study with UVR signatures (SBS7a-d) present are indicated, with a robust signature (>0.1) indicated in purple, those <0.1 in pink and those without SBS7a-d in black.

Even with the exclusion of the Krauthammer et al. study, the studies were still significantly different to each other (AM cohort, excluding Krauthammer Kruskall Wallis ANOVA *P* = 0.0007) or when the subtypes were (acral cohort, excluding Krauthammer Kruskall Wallis ANOVA *P* = 0.014; subungual cohort, excluding Krauthammer Kruskall Wallis ANOVA *P* = 0.028)

It was therefore decided the analysis of TMB would not be included in the meta-analysis, due to the significant differences between the cohort.

*Targeted Gene Studies (MSK-IMPACT GENIE)*

The American association for cancer research (AACR) project ‘genomics evidence neoplasia information exchange’ (GENIE) database [13] is hosted online on the cBioPortal platform (genie.cbioportal.org/) [11, 12]. The GENIE data was downloaded from the Synapse platform (synapse.org/). Using the release 8 public dataset, sample information was obtained from the file named ‘data_clinical_sample’ and the genomic data was extracted from ‘data_mutations_extended’ and ‘data_CNA’.

The OncoTree (oncotree.mskcc.org/) ontology code ‘ACRM’ for ‘acral melanoma’ was identified as a search term to identify relevant samples in the file ‘data_clinical_sample’. Searching through the ‘data_clinical_sample’ file identified 145 samples corresponding to the code ACRM. These samples represented a data compilation from multiple cancer centers, contributed by the Dana-Farber Cancer Institute (DFCI), Institut Gustave Roussy (GRCC), Johns Hopkins Sidney Kimmel Comprehensive Cancer Center (JHU), The University of Texas MD Anderson Cancer Center (MDA), Memorial Sloan Kettering Cancer Center (MSK), Netherlands Cancer Institute Center for Personalized Cancer Treatment (NKI), Princess Margaret Cancer Center (UHN) and Vanderbilt-Ingram Cancer Center (VICC). Each of the centres employed various experimental approaches and sequencing techniques to characterize the genomic data. For example, some centres only cover hotspot regions, while others capture sequencing data for entire coding exons, introns and promoters. Not all studies include a matched normal control. A comprehensive outline of the genomic characterization pipelines for each of the centres can be found in the synapse hyperlink under ‘data_guide.’

As our current study required both tumour and matched normal tissue sequenced, only MSK-IMPACT data was therefore included in our analysis. MSK-IMPACT includes coding exons, introns and promoters in their sequencing coverage. MSK-IMPACT have now applied three generations of sequencing panels, which varied by the numbers of genes covered in each panel (i.e. either 341, 410 or 468 genes; see Supplementary Table S1).

A total of 94 samples were identified in the MSK-IMPACT dataset for analysis. All sites were labelled as ACRM, and there was no distinction between acral palmar or plantar sites and subungual sites. Three patients had two tumour samples submitted for sequencing. As all three duplicate samples were derived from metastatic tumours with the same sequencing coverage, the first tumour of each duplicate was selected.

**Anatomical Classifications**

The studies included in our analyses cited a variety of anatomical sites (Supplementary Table S1). To homogenize these sites for the purpose of simplification and clarity, the reported primary sites were, where appropriate, re-categorized to represent an anatomical region, rather than a precise site. For example, primary site was broadly defined as ‘acral’ where the publication used terms such as “foot”, “sole”, “4^th^ toe”, etc. Similarly, a broad definition of “subungual” was given where the publication stated “toenail”, “thumbnail”, “Lt 3^rd^ finger nail”, etc. Primary sites were distinguished into “upper” and “lower” primary regions.

**Significantly Mutated Genes**

Variants were formatted into the mutation annotation format (MAF) using the Genome Analysis Toolkit Funcotator module v4.1.0.0. To identify significantly mutated genes in our dataset, MutSigCV version 1.3.5 was run via the GenePattern Public Server (<https://cloud.genepattern.org/>), including the MutSigCV pre-processing module. MutSigCV was run with the mutation type dictionary file provided by Broad and coverage and covariate files provided by GenePattern. Oncodrive FML (2.0.3; accessed via web interface) and OncodriveCLUST (0.4.1; accessed via *Maftools* package in RStudio) were run using default settings.

**Mutation Signatures**

For each sample the distribution of 96 single base substitutions (SBSs) with adjacent bases were calculated. Contribution from different mutation signatures were estimated by fitting the distribution as a linear combination of established COSMIC mutation signatures (version 3.0) [14] and minimizing the squared error. To avoid detection of insignificant contributions a backward selection was employed by calculating a p-value for each contribution and if the greatest p-value was greater than 0.05, that signature was removed and the analysis repeated until only significant contributions were included. The *p* value was estimated by repeating the analysis 1000 times, each time with different Poissonian noise added to the distribution of SBSs. The fraction of times a signature had zero contribution was used as estimated p-value.

**References**

1. Newell, F., et al., *Whole-genome sequencing of acral melanoma reveals genomic complexity and diversity.* Nat Commun, 2020. **11**(1): p. 5259.

2. Liang, W.S., et al., *Integrated genomic analyses reveal frequent TERT aberrations in acral melanoma.* Genome Res, 2017. **27**(4): p. 524-532.

3. Lim, Y., D. Yoon, and D.Y. Lee, *Novel Mutations Identified by Whole Exome Sequencing in Acral Melanoma.* J Am Acad Dermatol, 2020.

4. Hayward, N.K., et al., *Whole-genome landscapes of major melanoma subtypes.* Nature, 2017. **545**(7653): p. 175-180.

5. Berger, M.F., et al., *Melanoma genome sequencing reveals frequent PREX2 mutations.* Nature, 2012. **485**(7399): p. 502-6.

6. Furney, S.J., et al., *The mutational burden of acral melanoma revealed by whole-genome sequencing and comparative analysis.* Pigment Cell Melanoma Res, 2014. **27**(5): p. 835-8.

7. Hodis, E., et al., *A landscape of driver mutations in melanoma.* Cell, 2012. **150**(2): p. 251-63.

8. Krauthammer, M., et al., *Exome sequencing identifies recurrent somatic RAC1 mutations in melanoma.* Nat Genet, 2012. **44**(9): p. 1006-14.

9. Snyder, A., et al., *Genetic basis for clinical response to CTLA-4 blockade in melanoma.* N Engl J Med, 2014. **371**(23): p. 2189-2199.

10. Cancer Genome Atlas, N., *Genomic Classification of Cutaneous Melanoma.* Cell, 2015. **161**(7): p. 1681-96.

11. Cerami, E., et al., *The cBio cancer genomics portal: an open platform for exploring multidimensional cancer genomics data.* Cancer Discov, 2012. **2**(5): p. 401-4.

12. Gao, J., et al., *Integrative analysis of complex cancer genomics and clinical profiles using the cBioPortal.* Sci Signal, 2013. **6**(269): p. pl1.

13. *AACR Project GENIE: Powering Precision Medicine through an International Consortium.* Cancer Discovery, 2017. **7**(8): p. 818-831.

14. Alexandrov, L.B., et al., *Signatures of mutational processes in human cancer.* Nature, 2013. **500**(7463): p. 415-21.
